# Antimicrobial Photodynamic Therapy to Control Clinically Relevant Biofilm Infections

**DOI:** 10.3389/fmicb.2018.01299

**Published:** 2018-06-27

**Authors:** Xiaoqing Hu, Ying-Ying Huang, Yuguang Wang, Xiaoyuan Wang, Michael R. Hamblin

**Affiliations:** ^1^State Key Laboratory of Food Science and Technology, School of Biotechnology, Jiangnan University, Wuxi, China; ^2^International Joint Laboratory on Food Safety, Jiangnan University, Wuxi, China; ^3^The Wellman Center for Photomedicine, Massachusetts General Hospital, Boston, MA, United States; ^4^Department of Dermatology, Harvard Medical School, Boston, MA, United States; ^5^Center of Digital Dentistry, Peking University School and Hospital of Stomatology, Beijing, China; ^6^Harvard-MIT Division of Health Sciences and Technology, Cambridge, MA, United States

**Keywords:** photodynamic therapy, microbial biofilms, photosensitizer structure, biofilm-related infections, photochemical mechanisms, reactive oxygen species

## Abstract

Biofilm describes a microbially-derived sessile community in which microbial cells are firmly attached to the substratum and embedded in extracellular polymeric matrix. Microbial biofilms account for up to 80% of all bacterial and fungal infections in humans. Biofilm-associated pathogens are particularly resistant to antibiotic treatment, and thus novel antibiofilm approaches needed to be developed. Antimicrobial Photodynamic therapy (aPDT) had been recently proposed to combat clinically relevant biofilms such as dental biofilms, ventilator associated pneumonia, chronic wound infections, oral candidiasis, and chronic rhinosinusitis. aPDT uses non-toxic dyes called photosensitizers (PS), which can be excited by harmless visible light to produce reactive oxygen species (ROS). aPDT is a multi-stage process including topical PS administration, light irradiation, and interaction of the excited state with ambient oxygen. Numerous *in vitro* and *in vivo* aPDT studies have demonstrated biofilm-eradication or substantial reduction. ROS are produced upon photo-activation and attack adjacent targets, including proteins, lipids, and nucleic acids present within the biofilm matrix, on the cell surface and inside the microbial cells. Damage to non-specific targets leads to the destruction of both planktonic cells and biofilms. The review aims to summarize the progress of aPDT in destroying biofilms and the mechanisms mediated by ROS. Finally, a brief section provides suggestions for future research.

## Introduction

One major goal of modern clinical microbiology is to develop effective strategies to treat infections caused by microbial pathogen. Microbial biofilms account for up to 80% of all bacterial and fungal infections in humans (Høiby, 2017). A biofilm is a matrix-embedded microbial community attached to biological or non-biological surfaces (Hall-Stoodley et al., 2004). The microbial cells are embedded in extracellular polymeric substances (EPS) secreted by the cells. Compared to free-floating planktonic cells, biofilm cells exhibit altered physiological and metabolic states (Donlan, 2002). Biofilm formation is crucial for microbial survival in diverse and difficult environments (Hall-Stoodley et al., 2004), and in clinical situations, biofilms lead to difficult-to-eradicate infections by protecting microbes from attack by host defense systems and increasing microbial resistance to many antibiotics and biocides (Stewart, 2003). Biofilms are particularly involved in the pathogenesis of stubborn chronic infectious diseases (Wolcott and Ehrlich, 2008). Therefore, it is imperative to develop new approaches to fight against biofilm infections, and antimicrobial photodynamic therapy (aPDT) has been suggested as an efficient alternative approach. aPDT uses non-toxic photosensitizers (PS) to generate cytotoxic reactive oxygen species (ROS) upon irradiation by harmless visible light at specific wavelength. In this review, we critically summarize the killing effect of aPDT against clinically relevant biofilms *in vitro* and *in vivo* in the recent literature, and analyze the multiple underlying mechanisms of action.

## Biofilms and factors influencing their formation

Biofilm is a matrix-embedded microbial population enclosed by a self-secreted EPS, composed of polysaccharides, proteins, extracellular DNA, membrane vesicles, etc (Davey and O'Toole, 2000). Biofilm growth allows a protected lifestyle that allows pathogenic bacteria and fungi to survive in hostile environments (Hall-Stoodley et al., 2004), such as on medical devices and wound surfaces and on living tissues, inside animals or humans (Donlan, 2002). Microbes embedded in biofilm can tolerate 10–1000 times higher levels of antibiotics than their planktonic counterparts (Ceri et al., 1999). Biofilms have attracted increasing attention from researchers over the last 10 years. “*Biofilms and Microbiomes,”* a new Nature partner journal was launched recently (http://www.stm-publishing.com/open-access-nature-partner-journal-npj-biofilms-and-microbiomes/). The development process of biofilms involves the initial deposition of microbial cells onto a surface, reversible attachment to the substratum, irreversible attachment and colonization, aggregation and expansion, biofilm maturation, and finally biofilm dispersal and controlled detachment. A multidisciplinary review recently reported global understanding of the process of biofilm-formation (Karunakaran et al., 2011). Biofilm-determinants mainly belong to three categories: EPS (the external microbially produced environment), the microbial cell surface, and intracellular biomolecules (Karunakaran et al., 2011). During aPDT-mediated inactivation of biofilm, oxidative damage occurs in this order from external to internal. Thus, the main components of biofilms are described below.

### Components within the biofilm

#### Extracellular polymeric substances

EPS accounts for at least 90% by weight of the total biofilm content, and contributes to the structural complexity and strength of the biofilm (Chandra et al., 2001), thus EPS represents the first line of defense against diffusion of antibiotics, biocides, and also restricts the penetration of PS (Nett et al., 2010; Billings et al., 2013; Hengzhuang et al., 2013). The main components of EPS include a variety of carbohydrates, proteins, DNA, membrane vesicles, etc. These macromolecules are crucial for the initial adhesion of cells, since EPS can overcome the electrostatic repulsion between microbial cells and the substratum through a “polymer bridging” process and thereby ensure firm attachment or anchoring of microbial cells to the substratum (Neu et al., 2010). Moreover, some EPS molecules can affect the rheological properties and improve stability of the biofilm.

#### Cell-surface

The cell surface not only participates in the adherence of microbes to the substratum but also mediates the adhesion between neighboring microbial cells. There exist both similarities and significant differences between the structure and composition of cell walls and cytoplasmic membranes between gram-negative (G^−^) bacteria, gram-positive (G^+^) bacteria, and fungi (**Figure 2**). All of them have cytoplasmic membranes, which consist of a phospholipid bilayer containing embedded proteins. This membrane separates the cell interior from the external environment and is selectively permeable to inorganic ions and some organic molecules, and controls the uptake and excretion of metabolic substances. However, G^+^ bacteria possess a thick cell wall containing many layers of peptidoglycan and teichoic acids, while G^−^ bacteria have a relatively thin cell wall consisting of layers of peptidoglycan surrounded by a second outer membrane containing lipopolysaccharides (LPS) and lipoproteins. In contrast, *Candida albicans* (a fungal yeast) possesses a complex cell wall mainly containing chitin, glucan, and glycoproteins. Several research groups have investigated the properties of the bacterial cell surface in relation to colloidal stability and adhesion. According to a theory proposed by Derjaguin, Landau, Verwey, and Overbeek, the stability of colloidal particles is influenced by overall hydrophobicity and surface charge (Karunakaran et al., 2011). For example, Wang et al. reported that a mutant strain of *Cronobacter sakazakii* with an defective outer membrane had enhanced biofilm-forming ability, due to its higher surface hydrophobicity resulting from a defect in its coating of LPS (Wang et al., 2012).

#### Intracellular biomolecules

There are numerous biomolecules within microbial cell, carrying out countless biochemical reactions and metabolic processes that control the overall behavior of microbes. The microbial metabolism in the biofilm state differs markedly from that in the planktonic state, and these changes influence the physiology of pathogens and therefore can regulate biofilm development. For examples, by comparing nuclear magnetic resonance (NMR) metabolite profiling of methicillin-resistant *S. aureus* (MRSA) and methicillin-sensitive *S. aureus* (MSSA) in both biofilm and planktonic phenotypes, Ammons et al. found that the key differences included matrix deposition, and metabolism shifts, including selective amino acid uptake, lipid catabolism, butanediol fermentation, and a shift from metabolic energy production to assembly of cell wall components (Ammons et al., 2014). Alen et al. also discovered that the altered intracellular activity of *Neisseria meningitidis* in a biofilm environment contributed to enhanced survival, persistence and adaption to biofilm growth (van Alen et al., 2010). Furthermore, there also exist indirect effects on biofilm. For example, a change in the proteome can influence the structure and content of macromolecules expressed on the cell surface, and thence the EPS secreted into the matrix (Karunakaran et al., 2011), thereby affecting biofilm formation and cell aggregation.

### Biofilm-specific antibiotic resistance

The viscous matrix of biofilm can slow down diffusion of drugs and even act as a complete barrier against drug penetration (Singh et al., 2016). Moreover, for mixed-species biofilms, the various polymer can interact with each other, thereby further the increasing the viscosity and lowering drug penetration (Skillman et al., 1998). Moreover, microbial cells embedded in the deeper layers of the biofilm, change their metabolism to a state of near-dormancy, and became even less sensitive to antibiotics (Høiby et al., 2010). Furthermore, the high cell density within biofilms, encourages horizontal gene transfer and gene mutation, due to the physical proximity of the microbial cells and availability of extracellular DNA in the EPS (Molin and Tolker-Nielsen, 2003; Khemiri et al., 2016). The increase in genetic diversity within biofilms, facilitates adaption to antibiotics, and various drug-resistant variants have been reported to arise due to chromosomal mutation, induction of expression of latent genes, and exchange of genetic material between cells (Neu, 1992). For the reasons mentioned above, bacteria in biofilms are less susceptible to antimicrobial drugs, and bactericidal treatments (even aPDT) in comparison to their planktonic counterparts (Vickery et al., 2012).

Furthermore, some multi-species biofilms display lower susceptibility than mono-species biofilms. In a mixed-species biofilm of *S. mutans* and *Candida*, the hyphal formation of *Candida* was depressed due to its co-existence with *S. mutans* (Pereira-Cenci et al., 2008). Since it was easier for aPDT to kill hyphal forms of *Candida* compared to yeast forms (Chabrier-Roselló et al., 2008), the mixed-species biofilm displayed less susceptibility (Pereira-Cenci et al., 2008).

### Biofilm and quorum sensing (QS)

Biofilm formation of bacteria is a collective behavior involving bacterial populations embedded in a self-produced extracellular matrix. Moreover, the proximity of cells embedded in biofilms facilitates cell-to-cell communication. Thus, biofilm development are closely associated with QS, a cell–cell communication mechanism that senses population and orchestrate gene expression. QS describes the regulation of gene expression in response to fluctuations in population density, by which single-celled microorganisms (particularly bacteria) coordinate gene expression to suit their local population density and availability of nutrients. QS bacteria synthesize and release signaling molecules called “autoinducers,” that naturally increase in concentration as a function of cell density, until a threshold concentration of autoinducer is reached resulting in alteration in expression of related genes (Miller and Bassler, 2001). There are four different types of autoinducers reported to date, including N-acyl-l-homoserine lactone (AHL) in G^−^ bacteria, the autoinducing peptide in G^+^ bacteria, the autoinducer-2 in both G^−^ and G^+^ bacteria, and farnesol in *C. albicans* and other fungi. AHL in G^−^ bacteria has been shown to coordinate expression of genes responsible for diverse biological functions (Hentzer and Givskov, 2003; Linares et al., 2006). Compared to planktonic cells, bacterial cells within biofilms are much better protected from the attack by noxious agents. Consequently it is very difficult to control biofilm infections by antibiotics. The components within biofilm, as well as autoinducers, are targets of biofilm-controlling strategies including aPDT.

## Clinically relevant biofilms

Biofilms contribute to the majority of bacterial and fungal infections occurring in different anatomical regions of the body, such as urinary tract infections, catheter infections, middle-ear infections, gingivitis, caries, periodontitis, orthopedic implants, and many others (de Melo et al., 2013).

### Biofilm infections in different regions of the body

#### Oral biofilms and dental plaque

Bacterial biofilms on the tooth surface, also known as dental plaque, play a key role in the pathogenesis of periodontal diseases, endodontic infections, caries, and numerous other conditions extending beyond the oral cavity (Howlin et al., 2015). Dental pathogens including *Streptoccus mutans* (Takahashi and Nyvad, 2011), non-mutans acidogenic and aciduric bacteria (van Houte et al., 1996), such as streptococci, *Actinomyces* spp., lactobacilli, and *Bifidobacterium* spp. (van Ruyven et al., 2000). Among therapies designed to remove dental plaque, mechanical disruption and antimicrobial agents have been frequently employed, but the effectiveness is limited by amount of effort required and presence of resistant pathogens (Konopka and Goslinski, 2007). A high power laser showed some effectiveness, but caused thermal injuries to dental tissues (Nunes et al., 2011). In contrast, aPDT can remove oral biofilms on the teeth with negligible side-effects (Garcez et al., 2010; Rios et al., 2011; Vaziri et al., 2012; Yildirim et al., 2013; Muhammad et al., 2014; Zand et al., 2014), and thus in some clinical studies, aPDT has been used as an adjunctive treatment procedure to the conventional methods (Prasanth et al., 2014).

#### Chronic wound infections

Chronic wound infections lead to considerable morbidity and even amputation, and contribute to escalation in health care costs. Wound infections may progress from initial bacterial colonization followed by biofilm formation, which protects the bacteria from host defenses, while at the same time increasing resistance to conventional antibiotics (Edwards and Harding, 2004; Leaper et al., 2015). *Staphylococcus aureus* and coagulase-negative staphylococci are the most commonly isolated bacteria, and other common organisms include *Enterobacter cloacae, Enterococcus faecalis, Pseudomonas aeruginosa, Peptococcus magnus*, and anaerobic bacteria (Siddiqui and Bernstein, 2010). Most chronic wound pathogens are typical biofilm producers.

Currently, one of the most successful strategies for the management of biofilm-related conditions is physical removal of biofilm. Additionally, aPDT has been reported to be effective in eradicating wound infections caused by various pathogens (Wong et al., 2005; Zolfaghari et al., 2009; Lin et al., 2010).

#### Chronic rhinosinusitis (CRS)

Chronic rhinosinusitis (CRS) is one of the most common chronic conditions in the U.S., and a significant subpopulation of CRS patients is difficult to cure (Biel et al., 2013). MRSA and multidrug resistant *P. aeruginosa* have been isolated frequently from CRS patients (Wood et al., 2011; Li et al., 2012). Bacterial colonization and biofilm formation in the sinus cavity is implicated in the resistance mechanism. Recently, Biel et al reported that aPDT could effectively treat polymicrobial biofilms in a maxillary sinus cavity model (Biel et al., 2013).

#### Prosthetic joint infections (PJI)

As a major complication of total joint arthroplasty, prosthetic joint infections (PJI) are becoming a significant health concern. Although occurring at only a relatively low rate (ranging from 1 to 2%), PJI can lead to increased costs in health care (Sia et al., 2005). Bacterial biofilm that forms on prosthetic orthopedic implants is a common cause for PJI (Zimmerli et al., 2004). Highly virulent microorganisms such as *S. aureus* and G^−^ bacilli (such as *E. coli*) can participate in early infection, while coagulase-negative staphylococci and *Propionibacterium acnes* are responsible for some delayed infections, and *S. aureus*, streptococci, and G^−^ bacilli can contribute to late infections (Gbejuade et al., 2015). Recently, aPDT was reported as an efficient means of treating bacteria causing PJI (Briggs et al., 2018).

#### Ventilator associated pneumonia (VAP)

Ventilator associated pneumonia (VAP) is one of the most frequent hospital-acquired infections occurring in intubated patients, and can result in a mortality rate approaching 50% VAP is caused by a microbial biofilm forming on the inner surface of endotracheal tubes (ETT) (Azoulay et al., 2006). The most common species isolated from tracheal secretions and ETT-associated biofilm including *S. aureus, Enterococci, Enterobacteriaceae, P. aeruginosa, Acinetobacter baumannii*, and *Candida* species (Adair et al., 1999; Gil-Perotin et al., 2012). MB-aPDT can effectively eradicate polymicrobial biofilms in the ETT tube, which had long been recognized as a critical factor leading to VAP (Biel et al., 2011b).

## APDT and antimicrobial photodynamic inactivation

aPDT is a two-step technique employing a photosensitizer (PS) that is first administered systemically or topically to a confined area, followed by illumination with a specific wavelength of light that can excite the PS to cause production of cytotoxic ROS in the presence of ambient molecular oxygen. The burst of ROS produced during illumination can exert lethal effects on both cancer cells and/or microbial pathogens. aPDT has dual selectivity; the PS can be designed to be specific for cancers or infections, and the light can be spatially confined to the lesion area. The detailed features, types of PS and application in *in vitro* microbial cells are described below.

### PS structures and mechanisms

**Figure 1:**
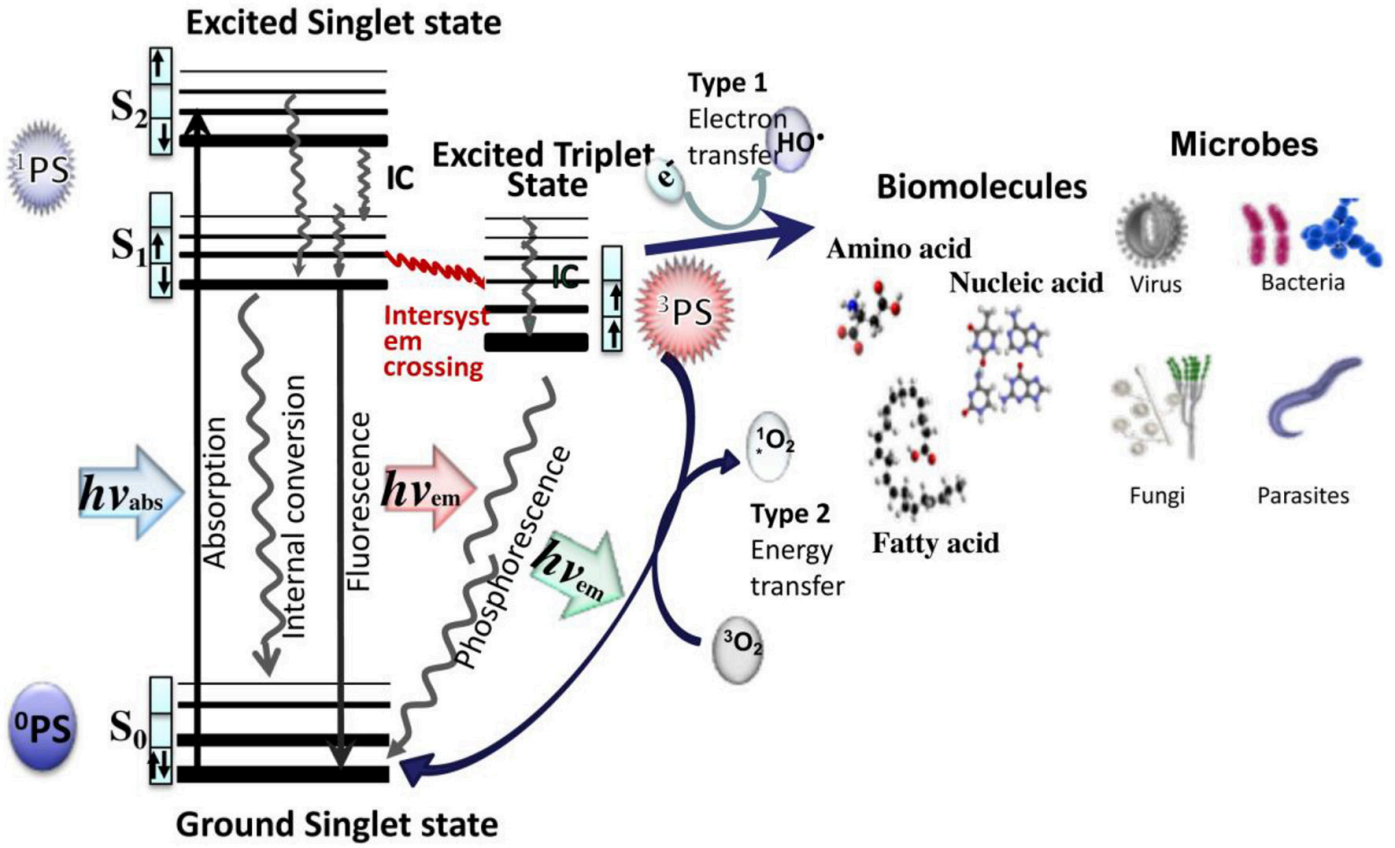
Jablonski diagram for ROS generation by aPDT via type I and type II photodynamic mechanisms. Both types of ROS can damage biomolecules and destroy or kill all known classes of pathogenic microorganisms.

aPDT employs specific-wavelength visible light to excite the PS. As shown in Figure 1, upon irradiation, the PS in its lowest energy level (ground singlet state, ^1^PS) is changed to the short-lived excited singlet state (^1^PS^*^), which can be converted to the long-lived excited triplet state, ^3^PS^*^. In presence of ambient oxygen, the triplet PS can undergo two types of chemical reactions: the type I mechanism is an transfer electrons to form toxic reactive oxygen species (such as superoxide, H_2_O_2_, and hydroxyl radicals, etc.) and the type II mechanism involves an energy transfer ground state triplet oxygen to produce highly reactive singlet oxygen (^1^O_2_). The two reactions can take place simultaneously.

PSs are usually highly conjugated unsaturated organic molecules with a large absorption coefficient in the visible spectrum and preferably in the long wavelength red near infrared region to ensure good tissue penetration of the light. However, not all dyes can undergo the intersystem crossing to produce the triplet state necessary for photochemical reactions to occur. Among PS structures commonly employed in aPDT (Table 1), acridine orange was the first used, and tetrapyrrole macrocycles such as porphyrins, chlorins, bacteriochlorins, and synthetic phthalocyanines can be highlighted. A number of non-tetrapyrrole dyes and natural compounds had also been used, such as rose bengal (RB), toluidine blue O (TBO), methylene blue (MB). As summarized by Abrahamse and Hamblin recently (Abrahamse and Hamblin, 2016), several PS such as HpD (haematoporphyrin derivative), Photofrin, PPIX (Protoporphyrin IX), Verteporfin (benzoporphyrin derivative), Radachlorin (now Bremachlorin), Fullerenes, Temoporfin, or Foscan (mtetrahydroxyphenylchlorin) had received clinical approval.

**Table 1:** PS chemical structures employed in aPDI and antibiofilm studies.

**PS**	**Structure**	**Manufacturer or source**	**Reference**
SAPYR	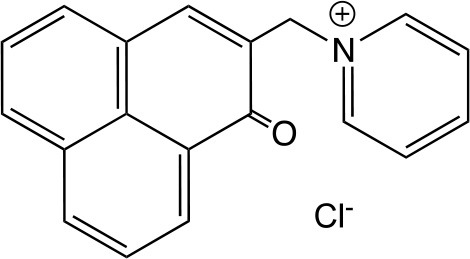	Synthesized by Cieplik et al. according to patent No. WO/2012/113860	Cieplik et al., 2013
Photogem	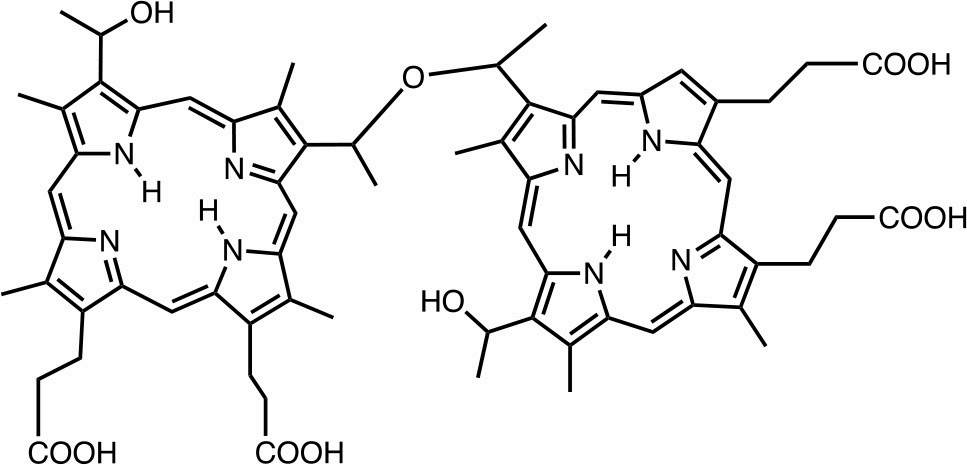	Tim Tec Corp., Newark, USA	Silva et al., 2014
PyP	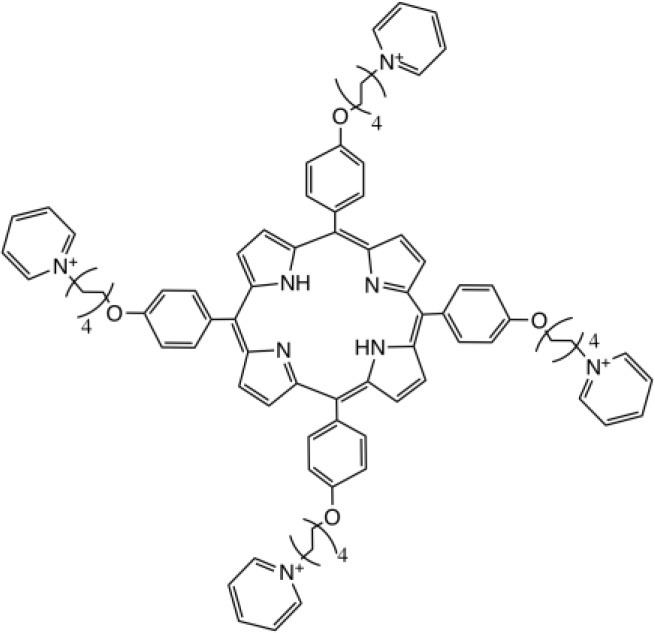	Synthesized by Prasanth et al.	Prasanth et al., 2014
RLP068/Cl	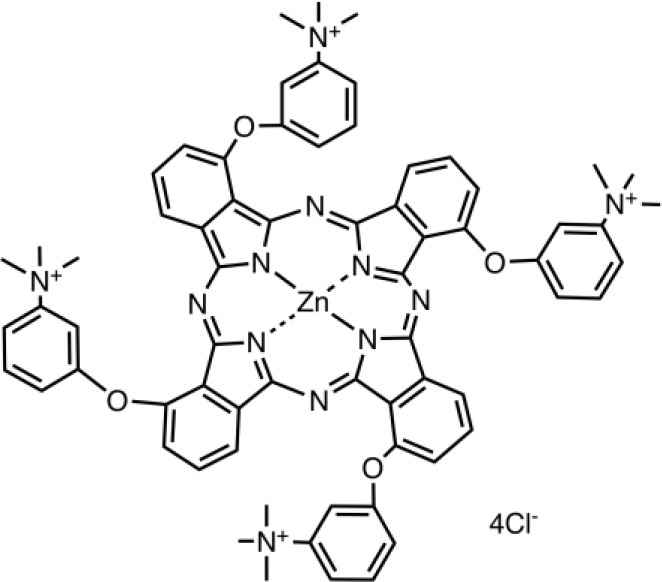	Molteni Therapeutics, Florence, Italy	Vassena et al., 2014
Cationic BODIPY	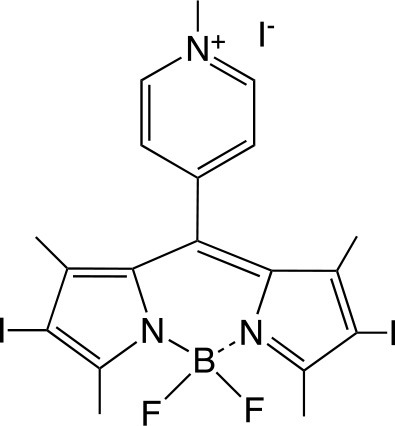	Prepared in ref. (Caruso et al., 2012)	Orlandi et al., 2015
Tetra-Py^+^-Me	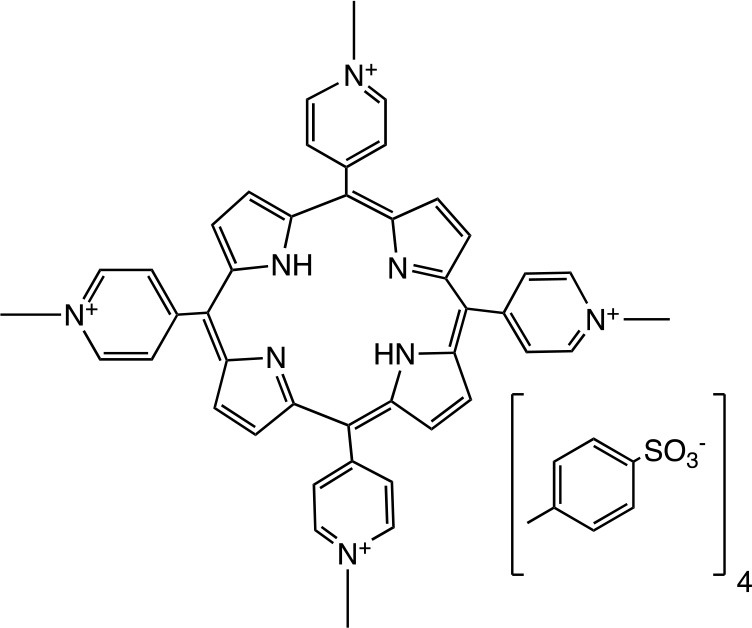	Prepared in refs (Carvalho et al., 2007, 2010; Gomes et al., 2011); Sigma-Aldrich Co. (St. Louis, MO)	Beirão et al., 2014
ClAlPc	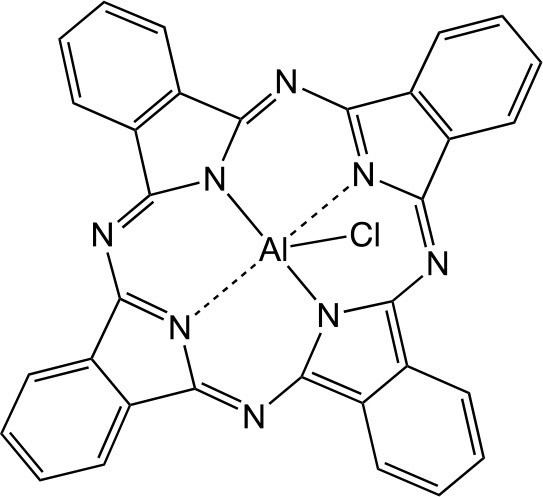	Sigma-Aldrich Co. (St. Louis, MO)	Ribeiro et al., 2013
FSc	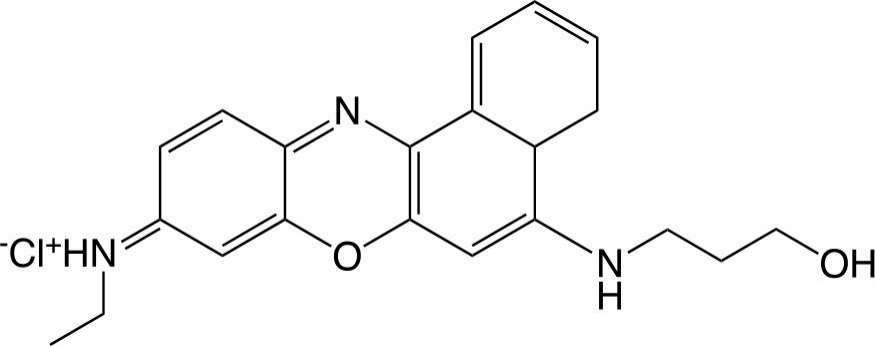	Synthesized in the Department of Chemistry of University of Minho (Frade et al., 2007)	Lopes D. et al., 2014
TBO	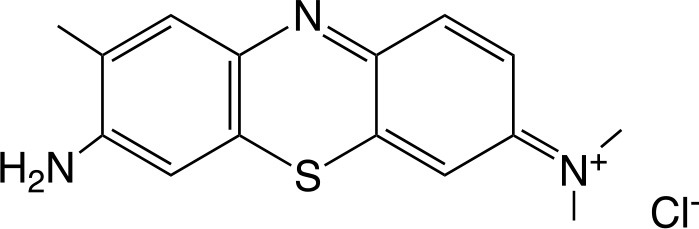	Sigma-Aldrich Co. (St. Louis, MO)	Tennert et al., 2014
MB	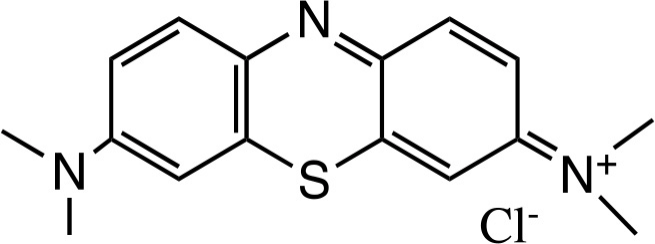	Sigma-Aldrich Co. (St. Louis, MO)	Garcez et al., 2013
Erythrosine	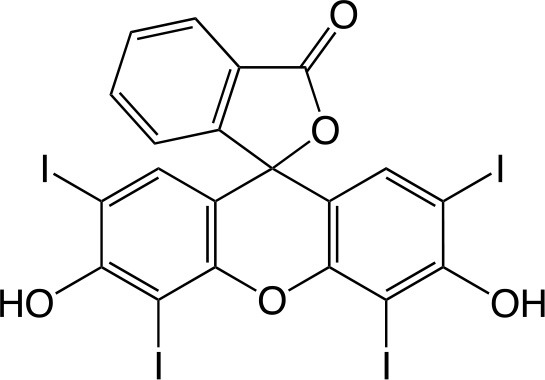	Sigma-Aldrich Co. (St. Louis, MO)	Cho et al., 2015
Curcumin	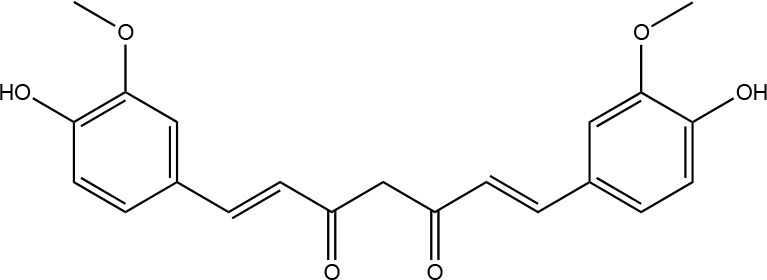	Sigma-Aldrich Co. (St. Louis, MO)	Manoil et al., 2014
XF-73	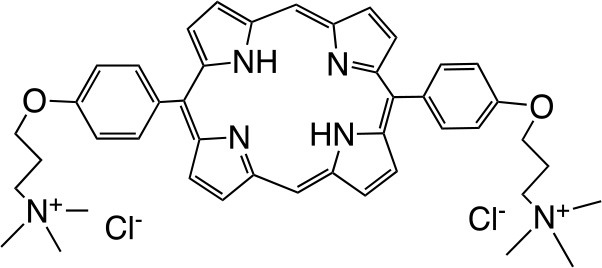	Synthesized by Destiny Pharma, Brighton, UK	Gonzales et al., 2013

**Figure 2:**
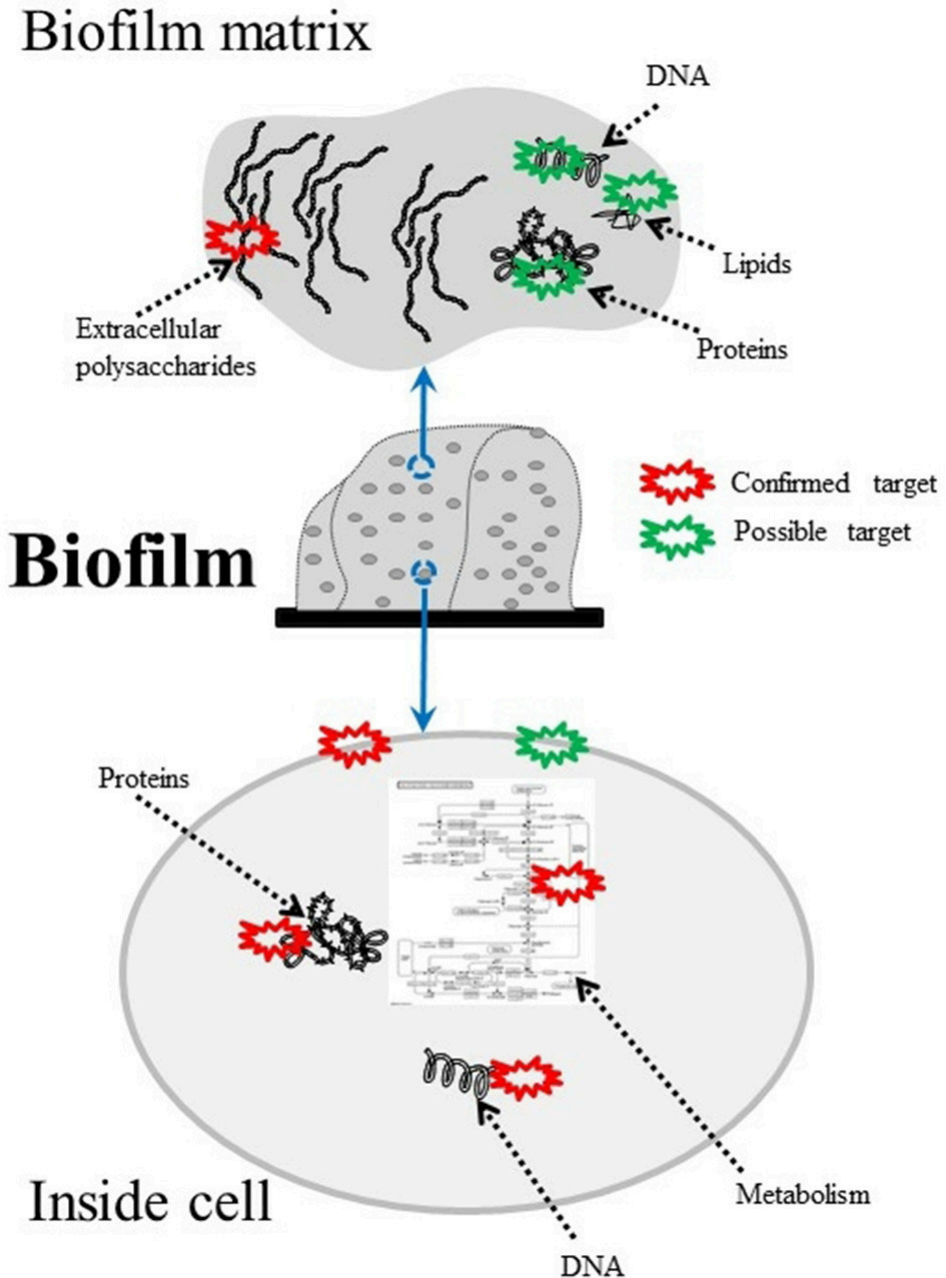
Composition of bacterial biofilms and possible aPDT targets. DNA, lipids (Alves et al., 2013a,b; Lopes D. et al., 2014), proteins (Konopka and Goslinski, 2007; Gracanin et al., 2009; Dosselli et al., 2012), DNA (Rabea et al., 2003; Lam et al., 2011), and polysaccharides (Beirão et al., 2014) can all be damaged by aPDT-generated ROS.

Furthermore in recent years, nanotechnology has been employed to improve PS performance (Chen et al., 2012; Khan et al., 2012). Figure 2 shows the targets of PS-generated ROS within microbial biofilms.

Here the PS molecules are chosen bind to the microbial cell surface, most often to the cytoplasmic membrane, so aPDI can take place. Thus, the attachment efficiency of PS has a great influence on the efficiency of aPDI. Considering the architectural differences in the cell surface structure, different microbial cells show different susceptibilities to aPDI depending on the PS chemical structure (George et al., 2009). In general, G^+^ bacteria and yeasts are easily affected by neutral and anionic PS, while G^−^ bacteria were not, because these species possess an additional asymmetric outer membrane containing numerous strongly negatively charged molecules such as LPS (Figure 2). This outer membrane reduces the permeability and weakens the attachment of neutral PS or repels anionic PS. To overcome the disadvantage, several strategies have been developed, such as synthesizing positively charged PS, coupling PS to other positively charged entities such as polyethylenimine, or pre-treating bacterial cells with Tris-EDTA or polymyxin B non-apetide (Nitzan et al., 1992).

### Susceptibility of biofilm forming pathogens to APDI

Up to now, a variety of pathogens have been isolated from different biofilm infections. As shown in Supplementary Table 1, aPDT has a broad-spectrum of activity against biofilm microrganisms including both naive and drug-resistant pathogens isolated from oral cavity, urinary tract infections, endocarditis, and biomaterial implant infections. These susceptible bacterial and fungal species cover the most frequently isolated pathogens, including G^−^ bacteria (*P. aeruginosa, E. coli, Fusobacterium nucleatum, Moraxella catarrhalis, Aggregatibacter actinomycetemcomitans*), G^+^ bacteria (*Enterococcus faecalis*, MRSA, MSSA, *Streptococcus* spp., *Actinomyces naeslundii*), and yeast (*C. albicans*).

## Investigative control strategies for biofilms

Before we look in detail at the ability of aPDT/aPDI to combat biofilms, we will present a brief survey of other innovative strategies that have been tested to eradicate biofilms.

### Inhibition of QS

QS coordinate the behavior depending on cell density, and thus interfering with QS signaling can decrease biofilm stability. Down-regulation of autoinducers or interference with their receptors is an approach that has been studied by several researchers (Davies et al., 1998; Geske et al., 2005; Ishida et al., 2007; Amara et al., 2009; O'Loughlin et al., 2013). The chemical reactivity of AHL was tuned by modifying the lactone head (Smith et al., 2003; Glansdorp et al., 2004; Ishida et al., 2007), and metabromo-thiolactone depressed biofilm formation by inhibiting QS receptor RhlR. Autoinducers operate via intracellular signal transduction by secondary messengers, such as cyclic diguanylate c-di-GMP. Thus, there existed a new approach to combat biofilms by modulating c-di-GMP signaling pathway (Römling et al., 2013). Ebselen could inhibit binding of c-di-GMP to allosteric sites, thereby inhibiting *P. aeruginosa* biofilm formation (Lieberman et al., 2014).

Some plant-derived natural products can interfere with QS, that might explain their use to treat microbial infections, such as *Quercus infectoria* Olivier (Chusri et al., 2012), *Curcuma longa* L. (Packiavathy et al., 2014), *Allium sativum* L. (Bjarnsholt et al., 2005), essential oils from *Cinnamomum verum* Pres (Niu et al., 2006), and *Syzygium aromaticum* (L.) Merrill & Perry (Khan et al., 2009), and methanol extract of *Kalanchoe blossfeldiana* (Sarkar et al., 2015). Other compounds targeting QS such as synthetic furanones (Wu et al., 2004), the fatty acid messenger *cis*-2-decenoic acid (Davies and Marques, 2009), and QS inhibitor “yd 47” have all displayed some efficacy against biofilms (Cevizci et al., 2015). Some proteins and peptides can also suppress biofilm by interfering with QS, such as RNA-III-inhibiting peptide (RIP) (Balaban et al., 2007), a competence-stimulating peptide (LoVetri and Madhyastha, 2010), and an analog of competence stimulating peptide (KBI-3221). Some combinations of QS inhibitors have exhibited synergistic efficacy, such as FS3 and daptomycin in treatment of staphylococcal biofilm (Cirioni et al., 2013).

### Enzymatic degradation of extracellular polymeric substances

These EPS-destructing enzymes include dispersin B (Kaplan et al., 2004; Itoh et al., 2005; Izano et al., 2008), an alginate lyase that can decompose polysaccharide (Alkawash et al., 2006; Alipour et al., 2009; Lamppa and Griswold, 2013), nucleases that can degrade extracellular DNA (also an important component of the biofilm matrix) (Okshevsky et al., 2015), and proteases such as proteinase K (Nicholson et al., 2013).

### Improvement of cleaning and disinfection of surfaces

The traditional cleaning and disinfection of surfaces could be improved by employing cold atmospheric plasma and reactive discharge gases (Traba and Liang, 2011; Pei et al., 2012); photocatalysis with TiO_2_ nanoparticles (Cieplik et al., 2013); nanoparticles of silver and gold (Adetunji et al., 2014); dense phase carbon dioxide (Mun et al., 2009); electrolyzed oxidizing water (Kim et al., 2001); antimicrobial mouth-rinse (Pedrazzi et al., 2014); vegetable oil (Brazil nut oil) and mineral oil (liquid petrolatum) (Filogoniô Cde et al., 2011); sanitizer treatments (Adetunji et al., 2014); a diode laser (de Oliveira Guaré et al., 2010); antibiofilm agent (El-Feky et al., 2009); UV-based oxidation process (Lakretz et al., 2011); and electrochemical biofilm control (Istanbullu et al., 2012).

### Other approaches

Bacteriophage therapy is effective in combating wound biofilm infections and implant- and catheter-related infections (Burrowes et al., 2011; Alemayehu et al., 2012; Seth et al., 2013; Soothill, 2013; Yilmaz et al., 2013; Chan and Abedon, 2015). Through blocking biogenesis of curli and type 1 pili (bacterial amyloids), two analogs of FN075 and BibC6 of ring-fused 2-pyridones were successfully applied to decrease biofilm formation (Cegelski et al., 2009). Some other antibiofilm compounds have been reported such as natural products secreted by marine sponges (Stowe et al., 2011), compounds based on the 2-aminobenzimidazole structure found in bromoageliferin and oroidin (Wright et al., 2014), and diterpenoid derivatives such as (-)-ageloxime-D from *Agelas* (Stowe et al., 2011). Other antibiofilm methods such as electrical treatments and ultrasound treatments have also been tested.

The antibiofilm strategies mentioned above aimed at specific and limited targets. In comparison, aPDT had multiple targets (described in section Underlying Anti-biofilm Mechanisms of aPDT), since the PS-generated ROS could non-specifically attack various molecules such as proteins, EPS, DNA and lipids. Therefore, it appeared to be promising and potentially applicable in very diverse contexts where PS and light can be easily delivered.

## The effect of APDT on biofilms *in vitro* and the underlying multiple mechanisms

To date, there is a growing body of literature indicating that aPDI represents an attractive approach to eradicate biofilms especially for treatment of recurrent and chronic biofilm infections. aPDI has multiple targets, and can not only efficiently kill the microbial cells themselves, but at the same time the ROS can weaken and degrade the matrix structure and the EPS by attacking numerous biomolecules. Therefore, aPDI appears to be promising and potentially applicable in many diverse contexts where PS and light can be easily delivered (Orlandi et al., 2015).

### G^−^ bacterial biofilms

As shown in Supplementary Table 1, most work has focused on *P. aeruginosa* and *A. actinomycetemcomitans*, and the highest bacterial killing efficiency was achieved with rates of about 7 log_10_ (Orlandi et al., 2015) and 6 log_10_ (Prasanth et al., 2014). aPDI using Tetra-Py^+^-Me was proposed to target polysaccharides in the biofilm matrix of *P. aeruginosa* first, and then inactivating the bacteria (Beirão et al., 2014). A more detailed mechanism of action on *P. aeruginosa* was proposed for RLP068/Cl (Vassena et al., 2014). This firstly targeted the cell wall and cell membrane by hydrophobic and electro-static interactions, then disrupted the external components on the cell surface through various oxidative reactions upon irradiation, and finally entered the cell by a self-promoted uptake process (Hamblin et al., 2002). Other susceptible G^−^ bacteria include *Fusobacterium nucleatum, E. coli*, and *Moraxella catarrhalis* clinical isolates. Especially, *M. catarrhalis* showed prominent morphological changes with visibly distorted cell membranes visualized by means of scanning electron microscopic (SEM) (Luke-Marshall et al., 2014).

### G^+^ bacterial biofilms

The most frequently employed G^+^ species have been *S. aureus, E. faecalis*, and *Streptococcus*, with highest killing efficiency reported to be 6.3 log_10_ (Beirão et al., 2014), *ca* 6 log_10_ (Prasanth et al., 2014), and *ca* 4.5 log_10_ (Chen et al., 2012), respectively. Especially, aPDT based on chitosan nanoparticles functionalized with rose-bengal (CSRBnp) decreased the viable bacteria of *E. faecalis*, and reduced biofilm thickness from 39.2 to 13.1 μm, through a mechanism where CSRBnp was propsed to adhere to the cell surface, permeabilize the cell membrane, and finaly lysed the cells (Shrestha et al., 2014). aPDT also showed antibiofilm activity for *A. naeslundii* and *Enterococcus* strains (Shrestha and Kishen, 2014).

### Yeast biofilms

*Candida* spp. can form biofilms on various implanted biomaterials such as pacemakers and intravascular catheters and thus can cause various clinical problems. Traditional treatment with antifungal agents is now facing challenges, and aPDI, either alone or in conjunction with other agents, has been proven to be effective. As shown in Supplementary Table 1, aPDT employing different PS (especially MB and TB) had been shown to control biofilms of *C. albicans* and *Candida parapsilosis*. 1 × 10^6^ cells were inoculated in 96-wells polystyrene microtiter plate to form biofilm, and aPDT treatment completely inactivated them (Lopes M. et al., 2014). The multiple mechanisms include inhibition of cell metabolism (Ribeiro et al., 2013), cell growth (Ribeiro et al., 2013), damage to the cytoplasmic membrane (Ribeiro et al., 2013), and increased cell permeability (Rosseti et al., 2014). When used as PS, *N*-(5-(3-hydroxypropylamino)-10-methyl-9*H*-benzo[*a*]phenoxazin-9-ylidene)ethanaminium chloride (FSc) and N-(5-(11-hydroxyundecylamino)-10-methyl-9H-benzo[a]phenoxazin-9-ylidene)ethanaminium chloride were absorbed by the biofilm matrix and cells. ROS were generated at these sites destroying the biofilm via oxidization of the matrix polysaccharide and damage to the cell wall (Lopes M. et al., 2014).

### Mixed-species biofilms

Almost all microbial biofilms occurring in nature are composed of various different species of microorganisms. Inter-species interactions, which can be synergistic interactions (metabolic cooperation) or antagonistic interactions (competition for nutritional resources), make mixed-species biofilms more complicated in architecture, and structure than mono-species biofilms. As shown in Supplementary Table 1, aPDT also showed some antibiofilm effects on mixed-species biofilms. When *S. mutans* ATCC25175 and *Lactobacillus acidophilus* ATCC ITAL-523 were cultivated together, the biofilm formed in 96-well plates could be completely removed by aPDT using a stock solution of curcumin and curcuminoids as the PS (Araújo et al., 2014). The underlying killing mechanism was similar to that for mono-species biofilms, including PS adhering to the cell surface, permeabilizing the cell membrane and finally lysing the cells (Cieplik et al., 2013). When the PS was functionalized by attaching it to bioactive nanoparticles, it increased the affinity to the cell membrane and allowed deeper penetration into the biofilm structure (Shrestha and Kishen, 2014).

### Underlying anti-biofilm mechanisms of aPDT

Based on the previous publications as mentioned below, it can be concluded that the anti-biofilm process of aPDT includes two steps. Firstly the PS initially binds to the biofilm matrix. In a few cases, the PS was totally sequestrated by EPS, while in other cases, the PS partially passed through EPS and penetrated further to contact the microbial cells. Some types of PS only bind to the cell surface, while other types of PS can pass through the cytoplasmic membrane and reach into the cellular cytoplasm and even enter organelles such as the nucleus in fungal cells. Wherever the PS was localized, ROS are generated upon illumination to initiate the second step, namely multitarget oxidative damage. Production of large quantities of ROS can overwhelm the antioxidant defenses of microbial cells. As soon as ROS is generated, it attacks a variety of adjacent molecules, including targets within the biofilm matrix (e.g., polysaccharides), on the cell surface (e.g., lipids), and inside the cells (e.g., proteins and DNA), resulting in the collapse of the biofilm matrix and disintegration of microbial cells. The generation of ROS has been extensively reviewed elsewhere (Melo et al., 2013), thus a detailed description is provided here with a focus on chemical reactions during the 2nd step.

Generally speaking, the burst of ROS (•OH and ^1^O_2_ are the two most commonly studied and damaging species) leads to oxidative damage of multiple non-specific targets, such as amino acids, nucleic acid bases, lipids, etc. The exact targets depend on the PS localization and abundance, since most ROS cannot travel very far before disappearing (Vatansever et al., 2013). Consequently, damage to proteins, DNA and membranes occurs, and cell death is induced. Furthermore, aPDT does not affect surrounding host cells or organs without PS. Based on the localization of PS in the biofilm, there exist three types of oxidative damage induced by aPDT as follows.

#### Biofilm matrix destruction

EPS-containing matrix (containing polysaccharides, proteins, hexosamine, uronic acid, and DNA) is the first line of defense against most environmental stress including aPDT. Moreover EPS matrix limits inward diffusion of the PS. For example, a slime-producing strain of *S. aureus* hampered PS uptake and showed lower sensitivity to aPDT than strains without slime (Gad et al., 2004). On the other hand, various components of the matrix can be attacked by ROS (Beirão et al., 2014). SEM imaging of *P. aeruginosa* or *E. faecalis* biofilms revealed damage to the architecture after MB-based aPDT (Garcez et al., 2013).

The most abundant constituent in matrix is polysaccharide, and its photodamage has attracted most attention (Garcez et al., 2013; Beirão et al., 2014). In 2014, Beirao et al. investigated photodynamic damage of polysaccharides within *P. aeruginosa* biofilms. PS Tetra-Py^+^-Me alone caused a 33% reduction of EPS content, while light alone did not. Because PS-matrix interactions in the absence of light likely affect the cohesiveness and stability of the EPS, and thereby can lead to polysaccharide detachment from the matrix (Beirão et al., 2014). However, when aPDT was conducted (after light delivery), the polysaccharide level was further reduced up to 80% (Beirão et al., 2014), which was crucial for the 2.8 log_10_ reduction in bacterial counts.

Besides polysaccharide, other constituents of the biofilm matrix such as proteins, lipids, DNA, and other components can easily be oxidized by ROS. It has been reported that macromolecules such as proteins and DNA in the microbial cytoplasm and lipids in the outer membrane could be irreversibly damaged by ROS (Konopka and Goslinski, 2007). Conjugation of PS to natural macromolecules such as chitosan also influenced EPS and contributed to the anti-biofilm effect (Shrestha and Kishen, 2012). The chitosan can intercalate with extracellular DNA and disrupt the biofilm structure (Upadya and Kishen, 2010).

### Damage to the cell surface

Up to now, little is known about the actual location of various PS in microbial cells and biofilms. Some PSs are tightly bound to the cell surface and can penetrate into the cells, such as poly-L-lysine chlorin(e6) conjugate (pL-ce6) (Merchat et al., 1996; Soukos et al., 1998), while other PSs may be only loosely bound and some not bound at all. Whether they are attached to cell surface or not, photoactivated PS result in damage to the cell membrane in most reports, via ROS oxidation (Shrestha et al., 2010; Luke-Marshall et al., 2014; Shrestha and Kishen, 2014).

Damage to the cell membrane has been observed by SEM after Photofrin-based aPDT treatment of a clinical *M. catarrhalis* biofilm (Luke-Marshall et al., 2014), and details of membrane damage were revealed after aPDT inactivation of MRSA biofilms using Ru(II) complexes as PS (Wood et al., 2006). Membrane damage causes morphological changes, such as appearance of bubble-like structures induced by osmotic fragility after cell wall damage (Wang et al., 2015). Besides, transmission electron microscope (TEM) clearly showed a discontinuous, detached and wrinkled cell profile, due to damage to the cytoplasmic membrane, which was similar to the shrunken cell envelope of *E. coli* seen after aPDT (Caminos et al., 2008). These changes always occurred at the polar regions of cells, which was attributed to their cardiolipin-rich domains (Mileykovskaya and Dowhan, 2000) and also to anionic phospholipids mostly located at polar regions (Oliver et al., 2014), where more intensive negative charge allowed a stronger affinity to the cationic Ru(II) complexes (Wang et al., 2015). Generally speaking, aPDT can irreversibly oxidize vital components of the cells as follows.

#### Oxidation of lipids

As soon as they are generated, ROS attack a variety of biomolecules, such as oxidizing unsaturated lipids, destructing DNA (particularly guanine bases) and inactivating proteins by attacking sulfur containing and aromatic amino acids. Recent studies highlighted lipid peroxidation upon photodynamic treatment of planktonic bacteria. Phosphatidylglycerol (PG) and cardiolipin (CL) are two major phospholipid components of *Staphylococcus warneri*. After aPDT treatment, the relative abundance of PG was increased while new oxidized species from CL such as hydroxyl and hydroperoxy derivatives appeared immediately (Alves et al., 2013a). The oxidation of lipids was also analyzed in *E. coli* (Alves et al., 2013b) by using a lipidomic approach. For *E. coli* ATCC 25922, aPDT induced appearance of lipid hydroperoxides, hydroxy and hydroperoxy derivatives, along with a decrease in unsaturated C16:1 and C18:1 species (Alves et al., 2013b). Melo et al investigated the oxidation of PE standards upon white light and cationic porphyrins, and identified photooxidation products of POPE (1-palmitoyl-2-oleoyl-sn-glycero-3-phosphoethanolamine, 16:0/18:1), PLPE (1-palmitoyl-2-linoleoyl-sn-glycero- 3-phosphoethanolamine, 16:0/18:2), and PAPE (1-palmitoyl-2-arachidonoyl-sn-glycero-3-phosphatidylcholine, 16:0/20:4) as hydroxy-, hydroperoxy- and keto- derivatives (Melo et al., 2013). Using 5 porphyrin derivatives with different molecular charges and analyzing lipid extracts from *E. coli*, it was revealed that content of lipid hydroperoxides depended on the PS charge and distribution (Lopes D. et al., 2014).

#### Change in permeability of the outer membrane

The primary consequence of damaging the outer membrane is a change in membrane permeability, responsible for death of the microbial cells, such as reported for TBO-mediated aPDT killing of *C. albicans* biofilms (Rosseti et al., 2014). Therefore, when some permeability-enhancing agents (such as chitosan and saponins) were combined with PS to mediate aPDT, the anti-biofilm efficiency could be further enhanced.

Coleman et al screened 12 saponins (biological detergents) and applied them together with PS to control *C. albicans* biofilms. The fungal susceptibility to aPDT was increased when combined with saponins, due to the formation of pores in the cell membrane that facilitated PS uptake (Coleman et al., 2010b). This observation also suggests that saponins could be an ideal supplement for conventional antifungal therapy (Coleman et al., 2010b). Some bioactive natural polymers such as chitosan can also permeabilize the cell surface of bacteria, due to the electrostatic interaction between ${\text{NH}}_{3}^{+}$ groups of chitosan acetate and phosphoryl groups of the phospholipids embedded in the cell membrane (Liu et al., 2004). The conjugation of PS with chitosan helped to enable more targeted action, increase PS uptake and improve antibiofilm efficacy (Rabea et al., 2003; Liu et al., 2004). By employing chitosan conjugated RB (CSRB), a higher killing effect on *E. faecalis* and *P. aeruginosa* biofilms compared to RB alone was obtained (Shrestha and Kishen, 2012).

The dramatic increase in permeability of outer membrane can improve PS uptake and stimulate metabolite leakage. Atomic force microscope (AFM) showed that RB-functionalized chitosan nanoparticles (CSRBnps) adhered to the cell surface of *E. faecalis*, and thereby resulted in pitting and disruption of cell surface (Shrestha and Kishen, 2014), which facilitated further penetration of CSRBnps (Shrestha and Kishen, 2014).

Another consequence of damage to the cell surface is interference with membrane function after a short aPDT treatment, such as reported for aPDT inactivation of *E. coli* biofilms (Caminos et al., 2008). This interference included inactivation of membrane transport systems (Hamblin and Hasan, 2004; Tavares et al., 2010), and other unknown mechanisms. In the latter case, damage to the cell surface of *Streptococcus mutans* was not serious; the bacteria did not die but could not replicate and grow, contributing to removal of *S. mutans* from dental plaque (Manoil et al., 2014).

#### Penetration and the self-promoted uptake pathway

In aPDT mediated by cationic PS such as the phthalocyanine RLP068/Cl, oxidative damage to the cell wall was followed by the self-promoted uptake pathway (Hamblin et al., 2002).

The self-promoted uptake pathway has been widely observed in polycationic antimicrobial agents in bacteria (Hancock and Bell, 1988). The initial step seems to occur at negatively-charged sites such as phosphate and carboxyl groups on lipopolysaccharide (LPS) molecules, which are the main lipid on the surface of G^−^ bacteria (Figure 2) and contribute to the relative impermeability of the outer membrane to neutral or anionic PSs (Nitzan et al., 1995). The non-covalent association between LPS and divalent cations (Ca^2+^ and Mg^2+^) is essential for the integrity for the G^−^ outer membrane (Leive, 1974), and cationic PS can weaken the attachment of adjacent LPS molecules by displacing these divalent cations (Minnock et al., 2000). Through the self-promoted uptake pathway, positively charged PS such as the cationic pyridinium zinc phthalocyanine (PPC) (Minnock et al., 2000) and the polycationic poly-L-lysine PS conjugate, pL-ce6 (Soukos et al., 1998) can gain access across the outer membrane of *E. coli*. pL-ce6 distorted the structure of outer membrane through the formation of channels (Soukos et al., 1998).

#### Intracellular damage

Some PS can pass through the cytoplasmic membrane, while other PS cannot immediately, but can arrive in the cytoplasm after irradiation. The intracellular PS can cause damage to countless biomolecules within the cytoplasm such as DNA, proteins, and lipids, which work together to interfere with vital microbial metabolic pathways and cell functions. Besides, for fungal cells, some organelles such as the nucleus and mitochondria can be damaged (Lam et al., 2011).

DNA damage is one crucial mechanism driving aPDI. aPDT leads to breaks in single-stranded and double-stranded DNA, and the disappearance of the super-coiled fraction of plasmid DNA in both G^+^ and G^−^ species (Bertoloni et al., 2000). A recent study revealed DNA cleavage at guanine residues caused by photoactivated Nile blue (NB) via electrostatic interaction with nucleic acids, which is thought to be an important mechanism in low oxygen environments (Hirakawa et al., 2014). On the other hand, with some less powerful PS, subtle non-lethal damage to DNA could be repaired by various DNA repair systems. The gene *uvrA* of *H. pylori* was implicated in the repair of DNA photodamage (Goosen and Moolenaar, 2008). However, for PS with powerful photodynamic effects, the burst of ROS induced significant DNA damage, which was impossible to be repaired. It was notable that Pc 4-mediated aPDT led to changes in nuclear morphology of *C. albicans*, which showed characteristics of apoptosis (Lam et al., 2011).

Proteins are another target, which are easily damaged, owing to their high abundance and rapid reaction rate with ROS. ROS can react with various amino acid residues in proteins resulting in loss of histidine residues, radical-induced cross links producing dityrosine moieties, introduction of carbonyl groups, formation of protein-centered alkyl, alkoxyl, alkylperoxyl, and ROO• radicals, and cleavage of peptide bonds (Gracanin et al., 2009). Especially, the sulfur-containing amino acids, cysteine, and methionine are particularly susceptible. The membrane proteomics of *S. aureus* after sub-lethal aPDT treatment using meso-tetra-4-N-methyl pyridyl-porphine revealed that expression of functional proteins involved in metabolic activities, such as oxidative stress defense, cell division, and sugar uptake were selectively affected (Dosselli et al., 2012).

**Figure 3:**
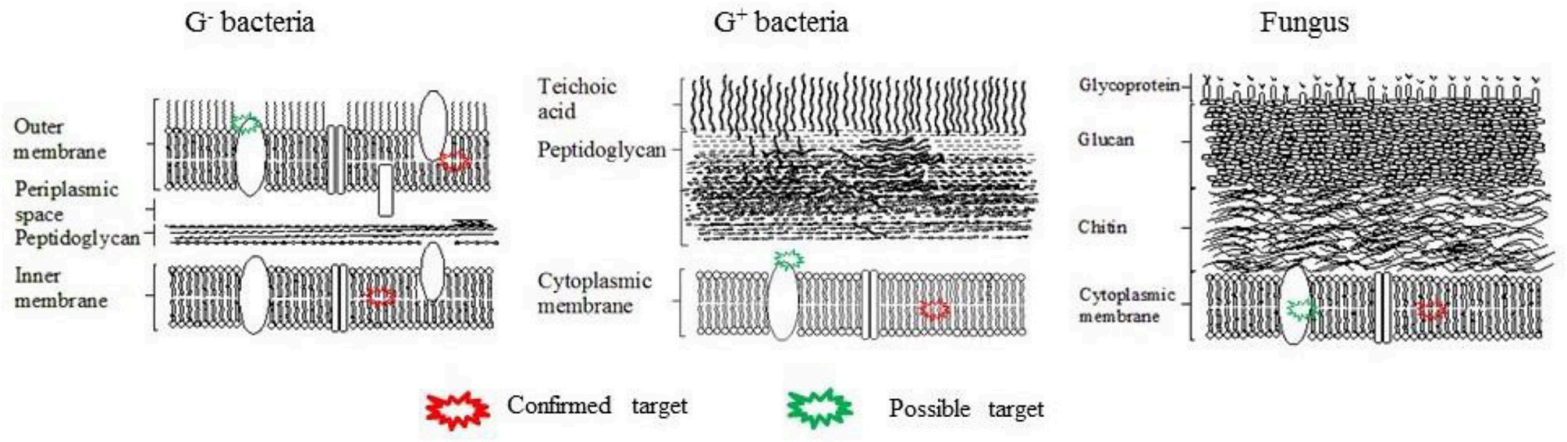
Cell wall structures of G^−^ bacteria, G^+^ bacteria and fungus and possible aPDT targets. The plasma membrane (Alves et al., 2013a,b; Melo et al., 2013; Lopes D. et al., 2014) and transmembrane proteins (Konopka and Goslinski, 2007). can be destroyed by aPDT-generated ROS.

It should be noted that due to the damage caused to protein and DNA, the cellular metabolism in biofilms can be significantly reduced by aPDT (Figure 3). de Aguiar Coletti et al. employed the 2,3-bis-(2-methoxy-4-nitro-5-sulfophenyl)-2H-tetrazolium-5-carboxanilide (XTT) reduction method to assess biofilm metabolic activity, because the biofilm need not be disrupted, and the intensity of the orange color of the formazan product is proportional to the metabolic activity. The results showed that aPDT led to decreases of 58 and 30% in cellular metabolism for *E. coli* and *S. aureus* biofilms respectively.

In addition to PS, chitosan can also interact with DNA and inhibit mRNA and protein synthesis (Rabea et al., 2003), partly contributing to the higher antibiofilm effect with chitosan-based RB (Shrestha and Kishen, 2012).

Because of the synergistic effect of cell surface and intracellular photo-damage, some phenotypic changes can be induced under sub-lethal aPDT treatment, such as reduced growth and lower glucose consumption in *S. aureus* (Dosselli et al., 2012), and inhibition of hyphae and blastoconidia formation of fungal species (Seneviratne et al., 2008). *C. albicans* biofilm is a complex mixture of blastoconidia, pseudohyphae and hyphae, and the co-existence of multiple cell forms indicates a mature biofilm (Seneviratne et al., 2008). Costa et al reported that erythrosine- and RB-mediated aPDT coupled with LED irradiation destroyed *C. albicans* biofilms by reducing blastoconidia and hyphae (Costa et al., 2012b). Another interesting finding was that 4-hydroxynonenal, a derivative of linoleic acid oxidation (Schneider et al., 2008), may serve as a cell stress-signaling factor that triggers stress responses (Dwivedi et al., 2007; Catala, 2009).

## Preclinical *in vivo* animal studies using APDT against biofilms

Before applying aPDT to clinical trials in humans, many *in vivo* studies have been carried out using animal models of various biofilm infections, including wounds, burns, middle-ear infections, endodontic infections, oral infections, osteomyelitis, nasal infections, *Helicobacter pylori*, leishmaniasis, tuberculosis, and different fungal infections (Table 1).

### Wounds and burns

Wounds and burns can be easily infected because the barrier function of the skin is disrupted. Burns are particularly susceptible to bacterial (especially multidrug resistant *S. aureus*) infections due to destruction of the cutaneous barrier (Cook, 1998).

For superficial wound infections where PS and light can be easily delivered, aPDT is thought to be particularly applicable to conquer bacterial biofilms. In a mouse model, Park et al assessed the aPDT effects on a mouse model of subcutaneous skin infection with bioluminescent *S. aureus* Xen29. After IV injection of chlorin e(6) and irradiation with a diode laser, lower bioluminescent signals were observed, suggesting growth inhibition. Furthermore, aPDT-treated mice had less neutrophilic infiltration and no massive bacterial colonies which the control group showed (Park et al., 2012). Nafee et al investigated aPDT using hypericin-loaded nanoparticles (HY-NP) in rats. aPDT treatment led to faster wound healing, better epithelialization, keratinization, and development of collagen fibers. The infection disappeared after 10 d, and an 80% reduction in the wound diameter was obtained (Nafee et al., 2013). Morimoto et al. evaluated the photodynamic effects of 5-aminolevulinic acid (5-ALA, precursor of protoporphyrin IX) on MRSA infection in mice. After intraperitoneal administration of 5-ALA, protoporphyrin IX accumulated in MRSA on the ulcer surface and aPDT exerted an anti-infective effect. 5-ALA-based aPDT accelerated wound healing and decreased the bacterial density on the ulcer surface (Morimoto et al., 2014).

A set of interesting models of localized infections in mouse skin has been investigated by Hamblin's group. Recently, skin abrasions infected by MRSA could be cured with aPDT mediated by PEI-ce6 conjugate (Dai et al., 2010). aPDT led to more than 3 log_10_ of bacterial reduction immediately but did not inhibit wound healing (Dai et al., 2010).

### Oral infections

The oral cavity provides an optimal environment for biofilm formation (Zaura et al., 2009). Up to now, three types of PS, erythrosine (Costa et al., 2012a), MB (Cernáková et al., 2015), and Photogem (Mima et al., 2010) have been employed to suppress oral biofilms in animal models.

Topical application of MB and laser irradiation using a diffusing fiber reduced *C. albicans* by 2.74 log_10_ steps in an immunosuppressed murine model (Teichert et al., 2002). Recently, Cernáková et al. also reported that photoactivated MB could totally eradicate biofilms of *C. parapsilosis* formed on the tongue of BALB/c female mice (Cernáková et al., 2015).

Photogem also displayed antibiofilm effects for *C. albicans* that was grown on the tongue of mice. For blue LED light, the maximal killing efficiency was achieved at 1.41 log_10_ after treatment, while for red LED light, a 1.59 log_10_ reduction rate was achieved, without any harm to the tongue tissue (Mima et al., 2010). Using erythrosine, aPDT reduced *C. albicans* in the lesions by 0.73 log_10_ and decreased its adherence to buccal epithelial cells by 35% without damaging adjacent tissue (Costa et al., 2012a).

### Osteomyelitis

Pathogenic bacterial and yeast infections in bone result in the formation of osteomyelitis. In a rat model, bioluminescent *S. aureus* biofilms were implanted into the rat tibial bone. ALA-aPDT was generally effective against not only *S. aureus* biofilms in bone, even against recurrent infection in some cases (Bisland and Burch, 2006), but also could destroy biofilms growing on implants in bone (Bisland et al., 2006).

### Nasal infection

The anterior nares are commonly infected by *S. aureus*, which could further spread to other anatomical regions after major surgery. Cale et al. evaluated the utility of aPDT for nasal MRSA decolonization using a custom nasal reservoir model. The MB-aPDT employed a 670 nm diode laser fiber and a disposable light diffusing tip (Street et al., 2009). aPDT eliminated sustained MRSA colonization on cultured human epithelial surfaces, and completely eradicated MRSA from the nose in humans with a treatment times less than 10 min (Street et al., 2009).

### Superficial fungal skin infection

Superficial fungal skin infections affect millions of people, where *C. albicans* and *Trichophyton rubrum* are the most frequently encountered fungal pathogens. Dai et al. employed a new MB-based model of aPDT in a mouse model of skin abrasion infected with *C. albicans* (Dai et al., 2011). aPDT initiated at 30 min or at 24 h post infection could reduce 95.4 and 97.4% cells of *C. albicans* in the skin abrasion wounds respectively.

Baltazar et al investigated the antifungal effect of TBO-aPDT in a mouse model of cutaneous dermatophytosis caused by *T. rubrum*. Topical application of a TBO gel was followed by red light irradiation given each day for 7 days. The fungal burden was reduced by 87%, and skin architecture was improved (Baltazar et al., 2015).

### Tuberculosis

Tuberculosis is caused by *Mycobacterium tuberculosis* infection, and the death rate in drug-resistant infections is among the highest for infectious diseases worldwide. aPDT had been applied to treat *Mycobacterium* infection in a mouse model, through injecting PS into the lesion and illumination using a fiber-optic. The subcutaneous granulomas formed from collagen gels were infected with *M. bovis*. Benzoporphyrin derivative (BPD), activated by a diode laser, led to 0.7 log_10_ reduction in viable bacterial numbers (O'Riordan et al., 2006), and another PS 5-ethylamino-9-diethylaminobenzo[*a*]phenoselenazinium chloride (EtNBSe) resulted in at least a 2 log_10_ decrease (O'Riordan et al., 2007).

### Otitis media

Otitis media (OM) is a very common childhood infection that responds poorly to standard antibiotics. Jung et al. investigated the preclinical effect of Photogem-aPDT. Two days after injection of *Streptococcus pneumoniae* or *Haemophilus influenzae* cells into the bullae of gerbil ears to produce a model of OM, Photogem was injected into the bullae, followed by transcanal irradiation with a 632-nm diode laser (Jung et al., 2009). aPDT was effective in eradicating *S. pneumoniae* in 87% of the infected bullae with OM, and *H. influenzae* in 50% (Jung et al., 2009).

## Clinical trials and patient studies

As shown in Table 2, aPDT has been employed in several clinical trials of localized biofilm infections in humans.

**Table 2:** Anti-biofilm aPDT studies in preclinical infections in animal models.

**Microbial pathogen**	**Animal models**	**PS and concentration**	**aPDT parameters**	**Reductions of CFU at log_10_ or other unit**	**Interval between microbial inoculation and aPDT**	**References**
**WOUNDS AND BURNS**						
Bioluminescent *S. aureus* Xen29	6-week-old male BALB/c mice	Chlorin e(6) [Ce(6)] (10 mg/kg mice)	664 nm, 100 J/cm^2^	Marked reduction (immediately); and complete reduction (5 d)	24 h	Park et al., 2012
MRSA ATCC 6538 and 7 clinical isolates	Female Wistar rats (200 ± 10 g, *n* = 12, Egypt)	HY-NPs (0.124 μM)	23.5 J/cm^2^	Disappearance (10 d after aPDT)	1 day	Nafee et al., 2013
MRSA ATCC 33591	8 to 10-week-old male C57BL/ksj db/db mice (Japan)	5-ALA (200 mg/kg)	410 nm, 164.5 mW/cm^2^, 50 J/cm^2^	Decrease	2 days	Morimoto et al., 2014
*S. aureus* strain 8325-4 transformed with *lux* operon	Male BALB/c mice (20–30 g)	PTMPP [500 μM (100 μl)]	635 ± 15 nm, 84 mW/cm^2^, 211 J/cm^2^, 42 min	Above 98%	24 h	Lambrechts et al., 2005
MRSA Xen31	Female BALB/c mice (6–8 weeks, 17–21 g)	Conjugate PEI–ce6 (ε400 nm = 150,000/Mcm)	660 ± 15 nm, 100 mW/cm^2^, 360 J/cm^2^	2.7 log_10_	30 min	Dai et al., 2010
*A. baumannii* ATCC BAA 747 transformed with *luxCDABE* operon	Adult female BALB/c mice (6–8 weeks; 17–21 g)	PEI-ce6 conjugate [800 to 900 μM (50 μl)]	660 nm, 100 mW/cm^2^, 240 J/cm^2^	Over 3 log_10_ (30 min); 1.7 log_10_ (1 day and 2 days)	30 min, 1 day, 2days	Dai et al., 2010
**ORAL PLAQUE**						
*C. albicans* ATCC 90028	Female Swiss mice (6 weeks)	Photogem [1000 mg/l (30 μl)]	455 nm, 305 J/cm^2^, 20 min	1.41 log_10_	4 days	Mima et al., 2010
*C. albicans* ATCC 90028	Female Swiss mice (6 weeks)	Photogem [500 mg/l (30 μl)]	630 nm, 305 J/cm^2^, 20 min	1.59 log_10_	4 days	Mima et al., 2010
*C. albicans* ATCC 18804	Adult male mice (30–60 g)	ER [400 μmol/l (50 μl)]	532 ± 10 nm, 90 mW, 14.34 J/cm^2^, 3 min	0.73 log_10_	24 h	Costa et al., 2012a
A clinical patient *C. albicans* isolate	Experimental beige nude mice with severe combined immunodeficiency disease	MB [500 μg/ml (0.05 ml)]	664 nm, 400 mW, 275 J/cm^2^, 687.5 s	2.74 log_10_ from oral cavity	4 weeks	Teichert et al., 2002
*C. parapsilosis* ATCC 22019	BALB/c female mice (7–8 weeks)	MB (1 mmol/l)	660 nm, 1.67 mW/cm^2^, 15 J/cm^2^, 2.5 h	Total prevention	72 h	Cernáková et al., 2015
**Osteomyelitis**						
Bioluminescent *S. aureus*	Rats (*rnu*/*rnu*, 150 g)	ALA (300 mg/kg)	635 ± 10 nm, 4.3 mW/cm^2^, 75 J/cm^2^, 4 h	Effective against biofilms in bone	16–21 days	Bisland and Burch, 2006
Bioluminescent *S. aureus* Xen29	Female Sprague-Dawley CD rats (250–300 g)	ALA (300 mg/kg)	635 ± 10 nm, 4.3 mW/cm^2^, 75 J/cm^2^, 4 h	Inhibition of biofilm implants in bone	10 days	Bisland et al., 2006
**SUPERFICIAL FUNGAL SKIN INFECTION**						
*C. albicans* CEC 749	Adult female BALB/c mice (7–8 weeks; 17–21 g)	New methylene blue [400 μM (50 μl)]	660 ± 15 nm, 120 J/cm^2^	Significant reduction	24 h	Dai et al., 2011
*T. rubrum* ATCC 28189	C57BL/6 mice	TB (0.2% in the gel)	630 nm, 42 J/cm^2^, 10 min	87%	7 days	Baltazar et al., 2015
**Tuberculosis**						
*Mycobacterium bovis* ATCC 35734	Male BALB/c mice (6–8 weeks, 20–25 g)	BPD (0.5 mg/kg)	690 nm, 65 mW/cm^2^, 60 J/cm^2^, 920 s	0.7 log_10_	N/A	O'Riordan et al., 2006
*Mycobacterium bovis* bacillus Calmette-Guerin (BCG) strain	Male BALB/c mice (6–8 weeks)	EtNBSe (5.25 mg/kg)	635 nm, 60-65 mW/cm^2^, 60 J/cm^2^, 920 s	Above 2 log_10_	N/A	O'Riordan et al., 2007
**Nasal infection**						
MRSA ATCC 33592	EpiDerm FT™ Full Thickness Skin Model	MB (0.01%)	670 nm, 96 J/cm^2^, 120 s	5.1 log_10_ (immediately) and 5.9 log_10_ (24 h after aPDT)	24, 48, 72 h	Street et al., 2009
**Otitis media**						
*S. pneumonia* ATCC 27336; and *H. influenza* ATCC 19418	30 Mongolian Gerbils	Photogem [1 mg/ml (20 μl)]	632 nm, 100 mW, 90 J, 15 min	killing *S. pneumonia* in 87.5% of infected bullae with OME, and killing *H. influenzae* in 50% of infected bullae with OME	2 days	Jung et al., 2009

*ALA, aminolevulinic acid; BPD, benzoporphyrin derivative; ER, erythrosine; EtNBSe, 5-ethylamino-9-diethylaminobenzo[a]phenoselenazinium chloride; HY-NPs, hypericin-laden nanoparticles; MB, methylene blue; PEI, polyethylenimine; Photogem: chemical structure shown in Table 2; PTMPP, meso-mono-phenyl-tri(N-methyl-4-pyridyl)-porphyrin; TB, toluidine blue*.

### Wound and ulcer infections

Two clinical trials were carried out for control of infections in non-healing chronic wounds (venous ulcers) and diabetic foot ulcers, using the phenothiazinium dye PP904 (3,7-bis(N,N-dibutylamino)-phenothiazinium bromide) (Morley et al., 2013) and tetracatiomic phthalocyanine RLP068 (Mannucci et al., 2014) as PS respectively. For 16 patients with chronic leg ulcers and 16 patients with diabetic foot ulcers with an ulcer duration longer than 3 months, PPA904 treatment for 15 min and red light irradiation at 50 J/cm^2^ were tolerated with no reports of pain and showed a reduction in bacterial load immediately post-treatment. Furthermore, half of the patients with actively treated chronic leg ulcers showed complete healing after 3 months, while only 12% of patients on placebo showed complete healing (Morley et al., 2013).

The RLP068 trial (Mannucci et al., 2014) was a randomized, double-blind, placebo-controlled phase IIa trial on 62 patients. aPDT was performed with topical application of three doses of RLP068 gel and 1 h later, red light irradiation with 60 J/cm^2^ was conducted. The results showed a reduction in microbial load dependent on dose, with the maximal killing efficiency (up to 3 log_10_) immediately post-illumination, and no safety issues.

### Dental infections

Clinical application of aPDT in dentistry have focused on treatment of infections arising from oral plaque biofilms. Root canals are commonly infected by bacteria, and the biofilm-forming *E. faecalis* is the most common. In order to sterilize root canals in patients, aPDT can be combined with the traditional approach of mechanical debridement for endodontic treatment (Tegos et al., 2006). aPDT was mediated by a conjugate between polyethylenimine and chlorin(e6) PEI-ce6 (Tegos et al., 2006) and illumination with a 660-nm laser. This approach improved the antibacterial effect evidenced by successive microbial sampling. Similarly, TBO-based aPDT and urea peroxide treatment achieved 82.59% reduction of viable bacteria via a synergistic effect (Pinheiro et al., 2009).

aPDT can be administered as an adjuvant after scaling and root planing (SRP) to treat patients with localized chronic periodontitis. In a study using competitive polymerase chain reaction (PCR) (Sigusch et al., 2010), a significant reduction of *F. nucleatum* DNA content was shown after 12 weeks, and significant reductions in reddening, bleeding on probing, and mean probing depth and a better level of clinical attachment were obtained. The adjuvant application of aPDT was shown to reduce intracellular DNA of *F. nucleatum* from 700–780 pg/ml (control group) to 50–180 pg/ml (aPDT group) and reduce periodontal inflammatory symptoms (Sigusch et al., 2010).

### Nasal decontamination

After preclinical testing of aPDT inactivation of nasal MRSA, a large clinical trial was conducted in Canada (Street et al., 2009). In combination with chlorhexidine gluconate, MB-aPDT led to 5.1 log_10_ reduction immediately and 5.9 log_10_ reduction after 24 h (Street et al., 2009). aPDT also reduced number of MRSA isolated from nasal swabs and number of post-operative surgical-site infections.

### *helicobacter pylori* infection

*H. pylori* (HP) can cause gastritis and gastroduodenal ulceration in humans and can even lead to stomach cancer. In 13 HP positive volunteers, a zone of gastric antrum was irradiated with laser or endoscopic white light after oral 5-ALA. Results showed that 4 h post-irradiation, the maximum eradication efficiency was achieved at 85% of biopsies for laser treatment group (58% HP-negative in the control), and 66% in white light irradiated group (33% HP-negative in the control) (Wilder-Smith et al., 2002).

Hamblin's group reported that *H. pylori* naturally accumulated large quantities of photoactive free porphyrins, and was thus susceptible to inactivation with violet light (405 nm) even without addition of exogenous PS (Hamblin et al., 2005), which led to the conduction of two clinical trials. The first trial used a small 1 cm diameter spot from a 405-nm laser to irradiate a part of the gastric antrum, with biopsies taken before and after to quantify *H. pylori* colony unit forming (CFU) (Ganz et al., 2005). Promising results from that trial led to a second trial with whole stomach illumination with 405 nm light (Lembo et al., 2009). The second trial showed that intra-gastric violet light phototherapy may represent a feasible and safe approach for eradicating *H. pylori*, especially in patients who had failed standard antibiotic treatment (Lembo et al., 2009).

### Possible side-effects

Up to now, aPDT has been shown to effectively eliminate bacteria and fungi with few side effects for the host tissues (Takasaki et al., 2009). The major side-effect after systemic administration of PS is the existence of a period of residual skin photosensitivity owing to PS accumulation. When activated by daylight, PS can cause burns, redness, and swelling until the drug is eliminated from the skin. These photosensitivity reactions could occur in minutes, therefore, exposure to bright light or direct sunlight must be strictly avoided. Other side-effects have included temporary coughing, trouble swallowing, stomach pain, or pain occurring at the area treated. There exist other potential side-effects that happen infrequently, such as allergic reaction, and change in liver function parameters (Vrouenraets et al., 2003; Kübler, 2005).

However the vast majority of the clinical applications for infections and biofilms, rely on topical or local administration of the PS, rather than systemic administration (intravenous or oral routes). Since the PS directly contact with the infected area, systemic absorption is thought to be minimal. Moreover, binding and uptake of the PS by the microbial cells and biofilms is rapid compared to the uptake by the surrounding host cells. Therefore, use of aPDI for localized infections usually requires a short drug-light interval (a few minutes) during which very little PS is expected to be taken up by host tissues, and subsequent light delivery will cause little damage to the tissue on which the biofilm is growing. The main issue with using aPDI for biofilm infections, is that after cessation of illumination, the production of actively antimicrobial ROS also ceases. Therefore, in animal models there have been problems with recurrence of microbial growth in the days following aPDT. This does not seem to have been such a big problem with clinical applications.

Along with development of novel PS, aPDT for infections will likely be more often clinically tested in the future and even receive more regulatory approvals as time goes on.

It is notable that in some preclinical *in vivo* animal studies (Jung et al., 2009; Baltazar et al., 2015) and clinical trials (Pinheiro et al., 2009), aPDT showed fungicidal efficiency or bactericidal efficiency below 3 log_10_CFU, thus much improvements of the aPDT method (alone and/or with conventional chemotherapy) are needed to increase the biofilm eradication rate.

Another point is that there are many light sources with a wide range of wavelengths had been employed in PDT, and no one light source is the optimum choice for different treatment regimes, even using the same PS (Coleman et al., 2010a; Agostinis et al., 2011). Both eye and skin injuries may occur from overexposure to high intensity light radiation, such as blue light can be toxic at high fluences (hundreds of J/cm^2^), thus it is important to avoid hazards from the light sources (Bullough, 2000). Fortunately, based on the publications in Tables 1, 2, no toxicity of light sources had been reported. While in the future, it should be investigated more deeply.

## Concluding remarks and future directions

Recently, the application of novel aPDT approaches in controlling biofilm infections had been reported both experimentally and clinically. Moreover, increasing evidence supports their effectiveness against other pathogens. In order to translate these findings into future clinical applications, several aspects deserve particular attention.

### Detailed mechanism of action

Up to now, the multiple targets of ROS-mediated aPDT have not been investigated systematically. aPDT attacks biofilm on three separate fronts: biofilm matrix, cell surface, and cytoplasm. The successful elimination of biofilms can be attributed to the synergistic effects of damage on these three fronts. Thus, a more holistic understanding of aPDT-susceptible targets is urgently needed. A detailed understanding of intracellular effects by means of transcriptomics, proteomics, and metabolomics would help to understand the underlying anti-biofilm mechanism of aPDT.

### More efficient delivery of PS and use of complementary interventions

aPDT treatment is initiated by the delivery of the PS. Fabrication of PS into suitable drug delivery forms may be critical for a high anti-biofilm efficacy. Various nanoparticles such as chitosan nanoparticles (Darabpour et al., 2016), silver nanoparticles (Zhou et al., 2017), and graphene oxide (Xie et al., 2017) have been shown to be useful, while the particle size and incubation time appear to be crucial for controlling *S. mutans* and *C. albicans* biofilm and require further optimization (Chen et al., 2012). Moreover, the PS drug concentration also requires to be optimized (Araújo et al., 2014; Luke-Marshall et al., 2014). If the concentration is too high it can act as an “optical shield” preventing the light from penetrating deeply enough. Some complementary compounds can be combined with aPDT to stimulate the anti-biofilm efficiency. For instance a QS inhibitor (QSI) would be a promising addition to PS. Some reported QSI such as diketopiperazines (de Carvalho and Abraham, 2012) have shown excellent potential for treatment of biofilm infections (Chung and Toh, 2014). Besides, efflux pump inhibitors could play a role, considering that some PS (such as phenothiazinium salts) have been shown to be substrates of microbial efflux pumps (Tegos and Hamblin, 2006). When verapamil, an efflux pump inhibitor, was combined with aPDT, a lower light dose was needed to get a higher reduction of cellular metabolism and reduced viability of bacterial biofilms, compared to that with PS alone, indicating the combination of aPDT with effux pump inhibitors may be more effective (de Aguiar Coletti et al., 2017).

### Possibility of inducing lower susceptibility to aPDT and higher virulence

Factors that affect the susceptibility of different microbial strains to aPDT need to be studied. After sub-lethal doses of aPDT, the biofilm-forming ability of *S. aureus* clinical isolates was increased (Kashef et al., 2013), which could make it less susceptible to a second application of aPDT. Blue light can activate production of colonic acid, a biofilm polysaccharide, produced under the influence of the biofilm-associated *bdm* gene in *E. coli* (Tschowri et al., 2009).

Other virulence factors such as a higher resistance to antibiotics could appear after aPDT (Kashef et al., 2013). Another virulence factor is modified LPS with altered expression of negatively charged phosphate groups (Renzi et al., 2015), and this change would likely weaken the binding of the PS. Besides, LPS can affect the polysaccharide chain itself (Díaz et al., 2015) to alter the hydrophobicity of the cell surface, which can influence the biofilm-forming ability of the cells. Future work is also needed to study effects on virulence factors after sub-lethal aPDT.

aPDT had been recently proposed to combat clinically relevant biofilms. The harmless visible light excited PS to generate ROS, which non-specifically attacked adjacent targets to damage both planktonic cells and biofilms. Numerous *in vitro* and *in vivo* aPDT studies demonstrated biofilm-eradication activities, and future work should be made to further improve efficiency in controlling biofilm.

## Author contributions

XH wrote the manuscript. Y-YH and YW helped to write the section of preclinical and clinical studies. XW helped to prepare the section of underlying mechanism. MH prepared Figure 1 and Table 3, rearranged and improved the manuscript.

**Table 3:** Anti-biofilm aPDT studies in human clinical trials.

**Microbial pathogen**	**Study subjects**	**PS (concentration)**	**aPDT parameter**	**Reductions of CFU log_10_ or other change**	**Reference**
**NON-HEALING ULCERS**					
*S. aureus, Corynebacterium striatum, P. aeruginosa, Alicaligenes faecalis, and E. faecalis*	62 patients aged C18 years	RLP068/Cl (0.50% in the gel)	689 ± 5 nm, 60 J/cm^2^, 500 s	3.00 ± 1.82 log_10_	Mannucci et al., 2014
MSSA and MRSA, *P. aeruginosa*, coliforms, beta-haemolytic *Streptococci*, and yeast	32 patients with chronic ulcers (16 venous leg ulcers, 16 diabetic foot ulcers)	PPA904 (500 μM)	570–670 nm, 50 J/cm^2^	2.5 log_10_ (immediately)	Morley et al., 2013
**DENTAL INFECTIONS**					
8 periodontal pathogens including *E. corrodens* FB69/36-26, *Actinomyces odontolyticus* P12-7, *Aggregatibacter actinomycetemcomitans* HIM 1039-8 Y4, *Fusobacterium nucleatum* ATCC 25586, *P. gingivalis* W381, clinical isolates of *P. micra, Atopobium rimae*, and *Slackia exigua*	six patients diagnosed with chronic periodontitis (CP)	Ce6 (C_34_H_36_N_4_O_6_, 100 mg/ml in 0.9% NaCl solution)	The continuous water-filtered spectrum covers 570–1400 nm (broad-band visCwIRA radiator), with local minima at 970, 1200, and 1430 nm, due to the absorption of water molecules. 200 mW/ cm^2^, 300 s	About 3.6 log_10_ for aerobic bacteria and 4 log_10_ for anaerobic bacteria	Al-Ahmad et al., 2016
*Fusobacterium nucleatum*	24 patients with localized chronic periodontitis (32–58 year old)	Phenothiazine chloride (N/A)	660 nm, 60 mW/cm^2^	Significant reduction of DNA concentration after 12 weeks	
**GASTRIC INFECTION**					
*H. pylori*	13 HP positive volunteers	ALA (20 mg/kg)	410 nm, 50 J/cm^2^	Greatly reduced	Wilder-Smith et al., 2002
*H. pylori*	10 patients (21-80 year old)	None	405 nm, 200 mW/cm^2^, 40 J/cm^2^	91%	Ganz et al., 2005
*H. pylori*	18 adults with *H. pylori* infection	None	408 nm, 15 min (5 patients), 30 min (5 patient), 45 min (7 patients), 60 min (1 patient)	Above 97% (in the antrum), above 95% (body), and above 86% (fundus)	(Lembo et al., 2009)

*Ce6, Chlorine e6; PPA904, 3,7-bis(N,N-dibutylamino) phenothiazin-5-ium bromide; RLP068/Cl, {1(4),8(11),15(18),22(25)-tetrakis[3-(N,N,Ntrimethylamonium)phenoxy]phthalocyaninato} zinc(II) chloride*.

### Conflict of interest statement

The authors declare that the research was conducted in the absence of any commercial or financial relationships that could be construed as a potential conflict of interest.
