# Supplementary material for: Antimicrobial Photodynamic Therapy to Control Clinically Relevant Biofilm Infections
Source: Front Microbiol. 2018 Jun 27;9:1299. doi: 10.3389/fmicb.2018.01299 (PMC6030385; doi:10.3389/fmicb.2018.01299)
Supplement: Supplementary file 1 [file Data_Sheet_1.PDF]

Supplementary Table 1. Anti-biofilm aPDT studies *in vitro*

Abbreviation for the photosensitizers: ALA: aminolevulinic acid; CS: chitosan; CSRBnp: RB-functionalized chitosan nanoparticles; ER: erythrosine; FSc: N-(5-(3-hydroxypropylamino)-10-methyl-9H-benzo[a]phenoxazin-9-ylidene)ethanaminium chloride; GD11: 2,6-diiodo-1,3,5,7-tetramethyl-8-(N-methyl-4-pyridyl)-4,4′-difluoroboradiazaindacene; MB: methylene blue; MG: malachite green; NE-ClAlPc: nanoemulsions of aluminum-chloride-phthalocyanine; RB: rose bengal; RLP068/Cl: {1(4),8(11),15(18),22(25)-tetrakis[3-(*N*,*N*,*N*trimethylamonium)phenoxy]phthalocyaninato} zinc(II) chloride; SAPYR: 2-((4-pyridinyl)methyl)-1Hphenalen-1-one chloride; PDZ: Photodithazine®, a chlorin e6 derivative; PPIX: Protoporphyrin IX; PyP: pyridinium-substituted porphyrin derivative; TB: toluidine blue; TBO: toluidine blue O; Tetra-Py$^+$-Me: 5,10,15,20-tetrakis(1-methylpyridinium-4-yl) porphyrin tetra-iodide; XF-73: 5,15-bis-[4-(3-trimethylammoniopropyloxy)-phenyl]-porphyrin

Microbial pathogen	PS (concentration)	Wavelength, light dose, irradiation time	Reductions of CFU in $\log_{10}$ or other units	Biological mechanism	Ref.
**G$^-$ bacteria**					
P. aeruginosa					
XEN5 transformed with *lux* operon	MB (60 µM)	660 nm, 9.6 J, 240 s	59.3%	N/A	(Garcez, et al., 2013)
ATCC 10145	MB (0.05%)	630 nm,150 mW, 30 J/cm^2, 200 s	A slight reduction	N/A	(de Freitas-Pontes, et al., 2014)
ATCC 27853	ER/CS nanoparticles (1.76 mg/ml)	540±5 nm, 22 mW/cm^2, 50 J/cm^2, *ca* 38 min	*ca* 2 $\log_{10}$	N/A	(Chen, et al., 2012)
PAE2 patient isolated strain	RLP068/Cl (50	690 nm, 120	*ca* 1.5 $\log_{10}$ in cell	PS targeted cell wall and membrane	(Vasse

(Milan, Italy)	μM)	mW/cm^2, 60 J/cm^2, 500 s	viability), and a reduction in biofilm volume	by hydrophobic and electro-static interactions, then disrupted external components on cell surface via oxidative reactions, and finally entered the cell by a self-promoted uptake pathway (Hamblin, et al., 2002)	na, et al., 2014)
Surfactant-resistant strain (António, et al., 2012)	Tetra-Py$^+$-Me (20 μM)	380-700 nm, 4.0 mW/cm^2, 21.6 J/cm^2, 90 min	2.8 $\log_{10}$	PS likely targeted polysaccharides in biofilm matrix at first, and then caused inactivation of bacteria	(Beirao, et al., 2014)
PAO1 and GFP-tagged strain PAO1	GD11 (2.5 μM)	400 nm, 48 mW/cm^2, 171 J/cm^2, 1 h	*ca* 7 $\log_{10}$	N/A	(Orlandi, et al., 2015)
A. actinomycetemcomitans					
ATCC 33384	ER (20 μM)	520-530 nm, 150 mW/cm^2, 9 J/cm^2, 30 s	88.9%	N/A	(Cho, et al., 2015)
ATCC 29523	MB (100 μM)	660±2 nm, 100 mW; 75 J/cm^2, 5 min	99.85%	N/A	(Alvarenga, et al., 2015)
Strain 8324 from DSMZ (Germany)	PyP (5 μM)	635 nm, 50 mW/cm^2, 60 J/cm^2, 20 min	*ca* 6 $\log_{10}$	N/A	(Prasanth, et al., 2014)
**Others**					
F. nucleatum ATCC 25586	PyP (5 μM)	635 nm, 50	*ca* 6 $\log_{10}$	N/A	(Prasa

		mW/cm^2, 20 min			nth, et al., 2014)
E. coli ATCC 25922	MB (200 µg/ml)	660 nm, 51 mW/cm^2, 44 J/cm^2, 860 s	3.4 log_{10} CFU/ml	combination of aPDT with verapamil significantly decreased cell metabolism	(de Aguiar Coletti, et al., 2017)
E. coli ATCC 11205	MB (0.05%)	630 nm, 150 mW, 30 J/cm^2	Significant reduction	N/A	(de Freitas-Pontes, et al., 2014)
Moraxella catarrhalis clinical isolates 7169, BC8, O35E, and CO72	Photofrin (125 µg/ml)	630 nm, 150 mW/cm^2, 225 J/cm^2, 1500 s	*ca* 3-4 log_{10}	SEM showed prominent distorted cell membranes	(Luke-Marshall, et al., 2014)
**G^+ bacteria**					
S. aureus					
ATCC 25923	MB (0.1% w/v)	635 ± 5 nm, 400 mW, 24 J, 60 s	1.55 log_{10}	SEM showed a clear reduction of the bacterial coating, with most cells showing ruptures of the bacterial wall, and small and scanty residual	(Giannelli, et al., 2017)

				colonies.	
ATCC 25923	MB (200 µg/ml)	660 nm, 51mW/cm^2, 22 J/cm^2, 430 s	3.38 log_{10} CFU/ml	Combination of aPDT with verapamil significantly decreases cell metabolism	(de Aguiar Coletti, et al., 2017)
ATCC 25923	MB (0.1 mg/ml)	660 nm; 40 mW, 7.2 J, 180 s	> 4 log_{10} on compact bone	N/A	(Rosa, et al., 2015b)
ATCC 25923	MB (0.1 mg/ml)	660 nm, 40 mW, 7.2 J, 180 s	3.06 log_{10} on cancellous bone specimens	N/A	(Rosa, et al., 2015a)
ATCC 25923	MG (0.1 mg/ml)	660 nm, 40 mW, 7.2 J, 180 s	4.46 log_{10} for specimens of compact bone	N/A	(Rosa, et al., 2015a)
ATCC 25923	MG (0.1 mg/ml)	660 nm, 40 mW, 3.6 J, 90 s	4 log_{10} reductions for the compact bone specimens	N/A	(Rosa, et al., 2014)
ATCC 25923	MG (0.1 mg/ml)	660 nm, 40 mW, 12 J, 300 s	3 log_{10} reductions for cancellous bone specimens	N/A	(Rosa, et al., 2014)
10 clinical strains recovered from acute and chronic wounds	Hypericin (0.5 µg/ml)	590 nm, 10 mW, 8 J/cm^2, 5 min	5.2-6.3 log_{10}	N/A	(Kashef, et al.,

					2015)
ATCC 25923	Hypericin (0.5 µg/ml)	590 nm, 10 mW, 16 J/cm^2, 10 min	5.7 $\log_{10}$	N/A	(Kashef, et al., 2015)
ATCC 700699	Tetra-Py$^+$-Me (20 µM)	380–700 nm, 4.0 mW/cm^2, 64.8 J/cm^2, 270 min	6.3 $\log_{10}$	PS likely targeted polysaccharides of the biofilm matrix at first, and caused inactivation of bacteria	(Beirao, et al., 2014)
MRSA ATCC 33592	MB (0.03%)	670 nm; 150 mW/cm^2, 8 min	3.1 $\log_{10}$ in the presence of 1.25 mM EDTA and 5% EtOH	N/A	(Biel, et al., 2013)
MRSA SAU-MRBP2	RLP068/Cl (50 µM)	690 nm, 120 mW/cm^2, 60 J/cm^2, 500 s	*ca* 1.5 $\log_{10}$ in cell viability and a reduction in biofilm volume	PS targeted cell wall and membrane, then disrupted external components on cell surface and finally entered cell by self-promoted uptake pathway (Hamblin, et al., 2002)	(Vassena, et al., 2014)
MRSA ATCC 67928, ATCC 68507, ATCC 35984	Tri-N-methylated analogue of zinc(II) phthalocyanine substituted with a triamino moiety (3.5 µM)	> 610 nm, 40 mW/cm^2, 48 J/cm^2, 20 min	Effective inhibition of biofilm	N/A	(Ke, et al., 2014)
E. faecalis					
E. faecalis ATCC 29212	MB (0.005 %)	670 nm, 280 mW, 8.4 J, 271 J/cm^2, 30 s ;	79%	N/A	(Lopez-Jimenez, et

					al., 2015)
E. faecalis	Indocyanine green (1 mg/mL)	810 nm, 200 mW, 30 s	99.12% reduction in colony count	N/A	(Afkhami, et al., 2017)
ATCC 1494	MB (60 μM)	660 nm, 9.6 J, 4 min	*ca* 88.7%	N/A	(Garcez, et al., 2013)
A strain	MB (N/A)	660 nm, 2 min	99.9%	N/A	(Yildirim, et al., 2013)
A clinical isolate	TB (13-15 mg/ml)	635 nm, 100 mW, 12 J, 120 s	92.7%	N/A	(Tennert, et al., 2014)
ATCC 29212	TB (0.1 mg/ml)	620–640 nm, 1000 mW, 106.4 J/cm^2, 30 s	Above 95 %	N/A	(Lopez-Jimenez, et al., 2015)
ATCC 29212	Chitosan conjugated RB (1mg/ml)	540 nm, 40 J/cm^2, 2 min	Inhibition of biofilm formation	N/A	(DaSilva, et al., 2013)

ATCC 29212	CSRBnp (0.3 mg/ml)	540 nm, 60 J/cm^2, 120 s	Reduction of viable bacteria, reduction of biofilm thickness from 39.2 μm to 13.1 μm (CSRBnp)	PS adhered to bacterial cell surface, permeabilized the membrane and lysed the cells	(Shrestha, et al., 2014)
ATCC 29212	SAPYR (100 μM)	410 nm, 600 mW/cm^2, 72 J/cm^2, 120 s	Above 5 log_{10}	PS can disrupt the structure of the biofilm, and kill bacteria	(Cieplik, et al., 2013)
A strain from Bulgarian National Bank for industrial microorganisms	5,10,15,20-Tetrakis[4-(8-pyridiniooctyloxy) phenyl]porphyrin tetrabromide (5 μM)	635 nm, 50 mW/cm^2, 60 J/cm^2, 20 min	*ca* 6 log_{10}	N/A	(Prasanth, et al., 2014)
5 clinical strains E2, E3, ER3/2s, OS16 and AA-OR34	Photogem® (0.25 mg/ml)	630 nm, 18.8 J/cm^2	Metabolic reduction approaching 100%	N/A	(Silva, et al., 2014)
Streptococcus					
S. mutans ATCC 35688	ER (5 μM)	455 ± 20 nm, 526 mW/cm^2, 95 J/cm^2, 36 J, 180 s	0.52 log_{10}	N/A	(Pereira, et al., 2013)
S. sanguinis ATCC 10556	ER (5 μM)	455 ± 20 nm, 526 mW/cm^2, 95 J/cm^2; 36 J, 180 s	0.88 log_{10}	N/A	(Pereira, et al., 2013)
S. mutans ATCC 25175	ER/chitosan nanoparticle (1 mg	540 ± 5 nm, 22 mW/cm^2, 50	*ca* 4.5 log_{10}	N/A	(Chen, et al.,

	ER/ml, and 0.5 mg chitosan /ml)	J/cm^2			2012)
S. mutans ATCC 35688	RB (5 µM)	455 ± 20 nm, 200 mW, 526 mW/cm^2; 36 J, 180 s	0.62 $\log_{10}$	N/A	(Pereira, et al., 2013)
S. sanguinis ATCC 10556	RB (5 µM)	455 ± 20 nm, 200 mW, 526 mW/cm^2, 36 J, 180 s	0.95 $\log_{10}$	N/A	(Pereira, et al., 2013)
S. mutans OMZ 918	Curcumin (40-60 µM)	360-550 nm, 450 mW/cm^2, 120 s	50% suppression of viability	N/A	(Manoil, et al., 2014)
S. mutans ATCC 27351	Porfimer sodium (125 µg/ml)	630 nm, 100 mW/cm^2, 30 J/cm^2, 5 min	Significant reduction	N/A	(Mang, et al., 2012)
**Others**					
8 *Enterococcus* strains from patients (Lower Silesia, Poland)	N/A	662 nm, 40 J/cm^2	Reduction of biofilms viability	N/A	(Dworniczek, et al., 2014)
A. naeslundii T14V	SAPYR (100 µM)	410 nm, 600 mW/cm^2, 72 J/cm^2, 120 s	*ca* 2 $\log_{10}$	PS adhered to cell surface, permeabilized cell membrane and lysed the cells	(Cieplik, et al., 2013)
**Fungal species**					
Candida					

C. albicans ATCC 001	MB (0.05%)	630 nm, 150 mW, 30 J/cm^2	A slight reduction	N/A	(de Freitas-Pontes, et al., 2014)
C. albicans ATCC 90028	PDZ (100 mg/L in 0.85% NaCl solution)	660 nm, 37.5 J/cm^2, 71 mW/cm^2, 540 s	1.2 log_{10}	N/A	(Alves, et al., 2017)
C. albicans ATCC 99901	PDZ (100 mg/L in 0.85% NaCl solution)	660 nm, 37.5 J/cm^2, 71 mW/cm^2, 540 s	1.14log_{10}	N/A	(Alves, et al., 2017)
C. albicans ATCC 36801 and ATCC 36802	MB (300 μM)	660 nm, 92 mW/cm^2, 26.3 J/cm^2, 10 J, 285 s	0.49 log_{10} and 2.34 log_{10}	It was maybe due to damage of cell wall structure	(Rossoni, et al., 2014)
Duo-species of *C. albicans*, *C. glabrata* and *C. tropicalis*	PDZ (150 mg/l)	660 nm, 37.5 J/cm^2, 540 s	1.0 log_{10} of viability	N/A	(Carmello, et al., 2017)
9 clinical *C. albicans* isolates and ATCC 18804	MB (300 μM)	660 nm, 13.78 mW/cm^2, 3.93 J/cm^2, 285 s	Drastic reduction	N/A	(Freire, et al., 2015)
C. albicans CEC 789	MB (90 μM)	660 nm, 100 mW, 108 J/tooth, 36 J/canal, 18 min/tooth; 6 min/canal	over 3.5 log_{10}	N/A	(Sabino, et al., 2014)

C. albicans SC5314, *C. parapsilosis* ATCC 22019, *C. parapsilosis* 16755/2, and *C. parapsilosis* 21922/1	MB (1.20-1.34 mmol/l)	660 nm, 75 mW/cm^2, 15 J/cm^2, 2.5 h	Total eradication of biofilm from the oral cavity	N/A	(Černáková, et al., 2015)
C. albicans ATCC 10231	TB (0.1 ng/ml)	630 nm, 68 mW, 21.47 J/cm^2, 2 min	62%	It was due to increase in the cell permeability	(Rosseti, et al., 2014)
C. albicans ATCC MYA-2876D	TBO (20 mM)	630±5 nm, 30 mW, 100 J/cm^2	Complete killing (chitosan incubation following aPDT)	Electron microscope showed that chitosan binded to the cell wall without irradiation, which likely disrupted cell cembrane	(Chien, et al., 2013)
C. albicans ATCC 90028	cationic NE-ClAlPc (N/A)	660 nm, 38.1 mW/cm^2, 100 J/cm^2	5% reduction on biofilm metabolism	PS inhibited fungal growth, damaged cytoplasmic membrane, and depressed cell metabolism	(Ribeiro, et al., 2013)
C. albicans ATCC 90028	Gold nanoparticles and MB conjugation (20 µg MB/ml)	660 nm,120 mW, 38.2 J/cm^2, 40 s	82.2%	N/A	(Khan, et al., 2012)
C. albicans ATCC 90028	Curcumin (40 µM)	455 nm, 22.0 mW/cm^2, 5.28 J/cm^2, 4 min	94%	N/A	(Andrade, et al., 2013)
C. albicans ATCC18804	RB (200 µM)	532 ± 10 nm, 90 mW, 42.63 J/cm^2, 16.2 J, 180 s	0.22 log_{10}	N/A	(Freire, et al., 2014)
C. albicans ATCC18804	Eosin Y (200 µM)	532 ± 10 nm, 90	0.45 log_{10}	N/A	(Freir

		mW, 42.63 J/cm^2, 16.2 J, 180 s			e, et al., 2014)
C. albicans MYA-2836^{TM}	ER/chitosan nanoparticle (1 mg ER/ml and 0.5 mg chitosan/ml)	540 ± 5 nm, 22 mW/cm^2, 50 J/cm^2	*ca* 3.5 $\log_{10}$	N/A	(Chen, et al., 2012)
C. albicans ATCC MYA-273	XF-73 (1 μM)	418 ± 20 nm, 13.4 mW/cm^2, 48.2 J/cm^2, 60 min	Over 5 $\log_{10}$	N/A	(Gonzales, et al., 2013)
C. albicans ATCC 10231	Tetra-Py$^+$-Me (5 μM)	380-700 nm, 4.0 mW/cm^2, 43.2 J/cm^2, 180 min	6.5 $\log_{10}$	PS likely targeted polysaccharides in biofilm matrix at first, and then inactivated bacteria	(Beirao, et al., 2014)
C. albicans ATCC 90028	FSc and N-(5-(11-hydroxyundecylamino)-10-methyl-9H-benzo[a]phenoxazin-9-ylidene)ethanaminium chloride (300 μM)	600 ± 2 nm, 10 mW/cm^2, 36 J/cm^2, 60 min	Complete cell inactivation	PS was absorbed by biofilm matrix and cells. ROS destroyed biofilm via oxidizing matrix polysaccharide and killing yeast via damaging cell wall	(Lopes, et al., 2014b)
**Multispecies biofilms**					
E. faecalis ATCC29212, *A. naeslundii* T14V	SAPYR (100 μM)	410 nm, 1360 ± 30 mW/cm^2, 120 s	*ca* 4 $\log_{10}$ (ATCC29212)	N/A	(Cieplik, et al., 2013)

S. oralis ATCC 35037, *P. intermedia* ATCC 25611, and *A. naeslundii* ATCC 12104	RB (10 mM)	540 nm, *ca* 41.67 mW/cm^2, 60 J/cm^2, 24 min	Reduction of biofilm thickness from 60.5 mm to 13.1 mm	N/A	(Shrestha and Kishen, 2014)
S. oralis ATCC 35037, *P. intermedia* ATCC 25611, and *A. naeslundii* ATCC 12104	CSRBnps (0.3 mg/ml)	540 nm, *ca* 41.67 mW/cm^2, 60 J/cm^2, 24 min	Reduction of biofilm thickness from 60.5 mm to 11.5 mm	N/A	(Shrestha and Kishen, 2014)
Multidrug-resistant *P. aeruginosa* (clinical isolate) and MRSA clinical isolate	MB (500 μg/ml)	664 nm, 150 mW/cm^2, 216 J/cm^2, 24 min (12 min on, 5 min off and 12 min on again)	*ca* 99.9%	N/A	(Biel, et al., 2011a)
P. aeruginosa ATCC 9027 and MRSA ATCC 33592	MB (0.03%)	670 nm, 150 mW/cm^2, 72 J/cm^2, 8 min	5 $\log_{10}$ (*P. aeruginosa*), and 3.1 $\log_{10}$ (MRSA)	N/A	(Biel, et al., 2013)
E. faecalis ATCC 29212, *A. naeslundii* T14V, and *F. nucleatum* ATCC 10953	SAPYR (100 μM)	410 nm, 600 ± 15 mW/ cm^2, 72 J/cm^2, 2 min	*ca* 5 $\log_{10}$ (ATCC 29212), and above 4 $\log_{10}$ (T14V)	PS adhered to cell surface, permeabilized cell membrane and lysed cells	(Cieplik, et al., 2013)
S. oralis ATCC 35037, *P. intermedia* ATCC 25611, and *A. naeslundii* ATCC 12104	CSRBnps (0.3 mg/ml)	540 nm, 60 J/cm^2, 120 s	Reduction	PS-functionalized bioactive nanoparticles increased affinity to cell membrane, and penetration into biofilm structure	(Shrestha and Kishe

					n, 2014)
Streptococcus gordonii ATCC 33399, *S. mutans* ATCC 25175, *F. nucleatum* ATCC 10953, *A. actinomycetemcomitans* ATCC 33384 and *P. gingivalis* ATCC 33277	Safranine O (5 mM)	532 nm, 0.5W, 50 J/ cm^2, 100 s	3 log_{10} suppression of bacteria	N/A	(Voos, et al., 2014)
S. aureus ATCC 700699 and *C. albicans* ATCC 10231	Tetra-Py^+-Me (20 μM)	380-700 nm, 4.0 mW/cm^2, 64.8 J/cm^2, 270 min	6.5 log_{10} (*S. aureus*), and 4.6 log_{10} (*C. albicans*)	PS likely targeted polysaccharides of biofilm matrix at first, and caused inactivation of bacteria in biofilms	(Beirao, et al., 2014)
S. mutans ATCC25175 and *L. acidophilus* ATCC ITAL-523	A stock solution of curcumin and curcuminoids (5 g/l)	450 nm, 19 mW/cm^2, 5.7 J/cm^2, 5 min	100% for 96-well plates and 69.4 % for dentine carious lesion	N/A	(Araújo, et al., 2014)
P. aeruginosa 27853 and *S. aureus* 29213	MB (500 μg/ml)	664 nm, 150 mW/cm^2, 60 J/cm^2, 400 s	*ca* 7 log_{10}	N/A	(Biel, et al., 2011a)
MSSA ATCC 25923, and MRSA ATCC 33591	Cationic chloro-aluminum phthalocyanine encapsulated in nanoemulsions (31.8 μM)	600 nm, 38.1 mW/cm^2, 50 J/cm^2, 26 min	Reductions of cell metabolism at 80% (susceptible strain) and 73% (resistant strain)	N/A	(Ribeiro, et al., 2015)
Clinical strains MRSA-1, MRSA-2, MRSA-3 and	Three Ru(II) polypyridine	457 nm, 40 mW/cm^2, 24	96.83%	PS destructed biofilm structure and bacterial cells	(Wang , et al.,

MRSA-4	complexes 1-3 (25 µM)	J/cm^2, 10 min			2015)
265 MRSA and 159 MSSA from hospital (Koszalin, Poland)	PPIX (25 µM)	620-780 nm, 102 mW/cm^2, 50 J/cm^2, 8 min and 10 s	High antibiofilm effectiveness	N/A	(Grinholc, et al., 2014)
S. gordonii ATCC 33399, *S. mutans* ATCC 25175, *F. nucleatum* ATCC 10953, *A. actinomycetemcomitans* ATCC 33384 and *P. gingivalis* ATCC 33277	Safranine O (10 µM)	532 nm, 0.5 w, 50 J/ cm^2, 100 s	3 log_{10}	N/A	(Voos, et al., 2014)
MRSA ATCC 43300, and methicillin-resistant *S epidermidis* MRSE 287	ALA (40 mM)	635 nm, 300 J/cm^2	*ca* 80% (ATCC 43300), and 90% (MRSE 287)	N/A	(Li, et al., 2013)
C. albicans ATCC90028, *Candida glabrata* ATCC2001, and *S. mutans* ATCC25175	PDZ (200 mg/ml)	660 nm, 71 mW/cm^2, 37.5 J/cm^2, 9 min	1.21 log_{10} (*C. albicans*), 1.19 log_{10} (*C. glabrata*), and 2.39 log_{10} (*S. mutans*)	N/A	(Quishida, et al., 2015)
15 clinical isolates (*C. albicans*, *C. tropicalis*, and *C. glabrata*)	PDZ (125 mg/l)	660 nm, 25 mW/cm^2, 37.5 J/cm^2, 25 min	0.9 log_{10} (*C. albicans*), 1.4 log_{10} (*C. tropicalis*) and 1.5 log_{10} (*C. glabrata*)	N/A	(Dovigo, et al., 2013)
S. gordonii ATCC 10558, *A. naeslundii* ATCC 12104, *F. nucleatum* ATCC 25586, *Campylobacter rectus* ATCC	Phenothiazine chloride (25 µl)	660 nm, 100 mW/cm^2, 180 s	2.44 log_{10}	N/A	(Eick, et al., 2017)

33238, *Fusobacterium alocis* ATCC 35896, *Eikenella corrodens* ATCC 23834, *P. intermedia* ATCC 25611, *Parvimonas micra* ATCC 33270, *P. gingivalis* ATCC 33277, *Tannerella forsythia* ATCC 43037, *Treponema denticola* ATCC 35405 and *A. actinomycetemcomitans* Y4					
S. aureus ATCC 700699, and *C. albicans* ATCC 10231	Tetra-Py^{+}-Me (20 µM)	380-700 nm, 4.0 mW/cm^2, 64.8 J/cm^2, 270 min	6.5 log_{10} (*S. aureus*) and 4.6 log_{10} (*C. albicans*)	PS likely targeted polysaccharides of biofilm matrix at first, and caused inactivation of bacteria in biofilms	(Beirao, et al., 2014)
Oral bacterial cells, such as *S. mutans, A. actinomycetemcomitans*, and *P. gingivalis*	$Au_{25}(Capt)_{18}$ clusters (500 µg/mL)	420-460 nm, 1 W/cm^2, 180 s	Destruction of biofilms	Application of Au clusters stimulated destruction of bacterial cell walls and inhibited biofilm formation	(Miyata, et al., 2017)
Bacteria within subgingival plaque samples from 10 patients	MB loaded poly(lactic-co-glycolic) nanoparticles (25 µg/ml)	660 nm, 100 mW/cm^2, 20 J/cm^2	0.69 log_{10}	N/A	(de Freitas, et al., 2016)
